# Human Memory B Cells Targeting *Staphylococcus aureus* Exotoxins Are Prevalent with Skin and Soft Tissue Infection

**DOI:** 10.1128/mBio.02125-17

**Published:** 2018-03-13

**Authors:** Adam J. Pelzek, Bo Shopsin, Emily E. Radke, Kayan Tam, Beatrix M. Ueberheide, David Fenyö, Stuart M. Brown, Qianhao Li, Ada Rubin, Yi Fulmer, William K. Chiang, David N. Hernandez, Hanane El Bannoudi, William E. Sause, Alexis Sommerfield, Isaac P. Thomsen, Andy O. Miller, Victor J. Torres, Gregg J. Silverman

**Affiliations:** a New York University School of Medicine, New York, New York, USA; b Department of Pediatrics, Vanderbilt University Medical Center, Nashville, Tennessee, USA; c Hospital for Special Surgery, New York, New York, USA; Trinity College Dublin; Harvard Medical School

**Keywords:** MRSA, *Staphylococcus aureus*, antibodies, host response, host-pathogen interactions, leukocidins, memory B cells, pore-forming toxins, skin and soft tissue infection (SSTI), superantigens

## Abstract

*Staphylococcus aureus* is a Gram-positive opportunistic pathogen that causes superficial and invasive infections in the hospital and community. High mortality from infection emphasizes the need for improved methods for prevention and treatment. Although *S. aureus* possesses an arsenal of virulence factors that contribute to evasion of host defenses, few studies have examined long-term humoral and B-cell responses. Adults with acute-phase skin and soft tissue infections were recruited; blood samples were obtained; and *S. aureus* isolates, including methicillin-resistant strains, were subjected to genomic sequence analysis. In comparisons of acute-phase sera with convalescent-phase sera, a minority (37.5%) of patients displayed 2-fold or greater increases in antibody titers against three or more *S. aureus* antigens, whereas nearly half exhibited no changes, despite the presence of toxin genes in most infecting strains. Moreover, enhanced antibody responses waned over time, which could reflect a defect in B-cell memory or long-lived plasma cells. However, memory B cells reactive with a range of *S. aureus* antigens were prevalent at both acute-phase and convalescent-phase time points. While some memory B cells exhibited toxin-specific binding, those cross-reactive with structurally related leucocidin subunits were dominant across patients, suggesting the targeting of conserved epitopes. Memory B-cell reactivity correlated with serum antibody levels for selected *S. aureus* exotoxins, suggesting a relationship between the cellular and humoral compartments. Overall, although there was no global defect in the representation of anti-*S. aureus* memory B cells, there was evidence of restrictions in the range of epitopes recognized, which may suggest potential therapeutic approaches for augmenting host defenses.

## IMPORTANCE

The contribution of B-cell memory and long-term antibody responses to host defenses against *S. aureus* exotoxins remains poorly understood. Our studies confirmed that infection did not commonly lead to enhanced long-term humoral responses. Whereas circulating memory B cells against *S. aureus* secreted exotoxins were prevalent, they were dominated by cross-reactivity with structurally related leucocidin subunits, consistent with recognition of conserved epitopes. These findings also provide the first evidence of a relationship between the reactivity of antistaphylococcal circulating memory B cells and serum antibody levels. In general, infection was not associated with a global defect in B-cell memory for *S. aureus* secreted factors, and responses were highly dominated by cross-reactivity to structurally related exotoxins, which arguably may alone be suboptimal in providing host defenses. Our studies illuminate aspects of the *S. aureus-*host relationship that may better inform strategies for the development of an effective protective vaccine.

## INTRODUCTION

*Staphylococcus aureus* is a Gram-positive opportunistic pathogen that has become the most common bacterial cause of serious, invasive, and fatal infections in both immunocompromised and healthy individuals, with more than 10,000 deaths per annum in the United States which also poses an immense economic burden (1–3). Skin and soft tissue infections (SSTI) due to *S. aureus* are most common, whereas pneumonia, osteomyelitis, endocarditis, and sepsis, although less common, account for greater morbidity and mortality (1). Antibiotic resistance in both hospital-acquired methicillin-resistant *S. aureus* (MRSA) and community-acquired MRSA (CA-MRSA) strains has increased the difficulty of treatment of these infections (4, 5). However, infection is not limited to immunologically naive hosts, as *S. aureus* chronically colonizes of 30% of adults, and most if not all humans have readily detectable antibody responses (6–9). The effects of prior *S. aureus* exposure via colonization on susceptibility to infection and immune competence are poorly understood.

Host defense from infection generally requires the development of a repertoire of antigen-selected clonally distributed T and B memory lymphocytes (10–12). Memory B cells arise from germinal center selection processes that often result in clones with high-affinity, class-switched receptors (13, 14), which can persist for years in the periphery (15). Memory B cells are poised to rapidly respond to antigenic rechallenge, with high-magnitude clonal expansions that generally display enhanced affinity for antigens and more-sustained responses (16). End-differentiated B cells secrete antibodies that contribute to host protection by aiding pathogen clearance and neutralization of toxins, which serve to hasten repair of tissue damage from inflammatory responses, as well as other functions (8, 17). The immunobiology of plasma cells, which maintain humoral protection, is largely intertwined with the development of memory B-cell recall responses (18).

As a consequence of the coevolutionary arms race between microbes and hosts, *S. aureus* is well equipped for immune evasion, possessing a formidable arsenal of immunomodulatory virulence factors as countermeasures that combat host immune responses, and has the capacity to constantly evolve its genetic makeup (4). *S. aureus* virulence factors include superantigens and pore-forming toxins (such as hemolysins and leucocidins) that are capable of targeting a range of innate and adaptive immune cells, and proteomic and transcriptional studies suggest enhanced expression of these factors during infection (19–22).

In mice, intravenous infection with sublethal doses of live *S. aureus* confers protection that correlates with the strength of the postchallenge antibody response to staphylococcal antigens (23). However, murine models of *S. aureus* infection have been unreliable predictors of the success of experimental *S. aureus* vaccines in clinical trials (8, 24, 25), and mice with a competent B-cell compartment fare worse in systemic infection models than do mice genetically engineered to be devoid of B cells (26–29). These findings suggest that factors produced during *S. aureus* infection have poorly understood effects on host defenses. These perspectives may be important for efforts to develop a prophylactic *S. aureus* vaccine in humans, which have heretofore been uniformly unsuccessful (30).

Virulence factors produced by *S. aureus* are postulated to affect adaptive immune responses and susceptibility to the recurrence of infection. While prior colonization or infection may induce some level of immune protection (9, 31), such exposure does not uniformly result in protection from future or recurrent clinical infections (32–34). Currently, the range of B-cell epitopes targeted following *S. aureus* exposure and infection remains unclear (35–37); therefore, identification of the microbial and host factors that contribute to human protective immunity against *S. aureus* remains a challenge.

To survey for potential defects in humoral and cellular responses to *S. aureus* antigens, we recruited patients with culture-confirmed *S. aureus* SSTIs and explored longitudinal serological antigen-reactive antibody titers, as well as anti-*S. aureus* memory B-cell responses, using a panel of *S. aureus* and control antigens. Our report provides an in-depth dissection of the immune recognition of a range of *S. aureus* exotoxins by circulating antibodies and memory B cells in the context of active infection and convalescence.

## RESULTS

### Characterization of *S. aureus* isolates from patients with SSTI.

For our studies of clinical *S. aureus* infection, we enrolled 115 ethnically and demographically diverse patients with primary SSTIs at two geographically adjacent clinical sites in New York, NY, among whom 54 patients (47.0%) were *S. aureus* culture positive (see Table S1 and S2 in the supplemental material), whereas 36 (31.3%) grew other microbes. Of the 54 *S. aureus*-infected patients, 59.3% had MRSA infections and 40.7% had methicillin-resistant *S. aureus* (MSSA) infections (Table S2), which are comparable to the results seen in earlier studies demonstrating a high frequency of community-acquired MRSA (CA-MRSA) among SSTI patients (see, for example, reference 38). Indeed, CA-MRSA USA300 (*spa* type 1/t008, positive for staphylococcal cassette chromosome *mec* type IV [SCCmec IV], *pvl*, and the arginine catabolic mobile element [ACME]) was the most frequent strain lineage, representing isolates from 24.5% of patients (Table S2). Sixty-three different strain types (61.9% MRSA and 38.1% MSSA) were recovered from the infected sites of 53 *S. aureus* patients, including 6 patients (11.1%) with mixed infections due to multiple clone types. The *pvl* gene (which encodes leucocidin SF [LukSF]) was present in 87% of infecting strains (Table S2) and was pervasive in both MRSA and MSSA strains, consistent with reports of other recently described SSTI cohorts (39). The *pvl*-negative infecting strains were isolated from patients with comorbid conditions (four with diabetes and three with unspecified diseases), suggesting that these may represent misclassification of secondary infections.

10.1128/mBio.02125-17.7 TABLE S1  *S. aureus* skin and soft tissue infection patient data. Clinical observations, treatment details, and patient-reported demographic and lifestyle information are provided for the cohort of *S. aureus* skin and soft tissue infection (SSTI) patients enrolled for this study. Download TABLE S1, PDF file, 0.3 MB. Copyright © 2018 Pelzek et al. 2018 Pelzek et al. This content is distributed under the terms of the Creative Commons Attribution 4.0 International license.

10.1128/mBio.02125-17.8 TABLE S2  Infecting and colonizing *S. aureus* isolates from SSTI patients. Clinical (*n* = 54 patients, *n* = 63 isolates) and colonizing (*n* = 25 patients, *n* = 29 isolates) isolates were assessed for antibiotic resistance profile (methicillin-resistant *S. aureus*, MRSA; methicillin-sensitive *S. aureus*, MSSA), presence or absence of the *pvl* gene, and *spa* typing result. USA300 strains were confirmed as *spa* type 1/t008 and were positive for SCCmec IV, *pvl*, and ACME. Download TABLE S2, PDF file, 0.1 MB. Copyright © 2018 Pelzek et al. 2018 Pelzek et al. This content is distributed under the terms of the Creative Commons Attribution 4.0 International license.

Of the 54 patients infected with *S. aureus*, 25 (46.3%) were colonized in the nares or groin at presentation. Among these, 64.0% of the colonizing strains were the same as the infecting strain based on *spa* typing and genome sequence analysis (Table S2). Culture-negative patients were found to be colonized with *S. aureus* only rarely (8.2%, 5/61). These results are consistent with the known association between colonization and infection by *S. aureus* (40).

We completed whole-genome sequencing for infecting *S. aureus* isolates from 92.5% of patients. The sequenced genomes enabled us to characterize each strain for the presence and polymorphism of genes encoding key *S. aureus* virulence factors, with a focus on staphylococcal leucocidins and superantigens (see Fig. S3 in the supplemental material). These strains contained the core genome-associated exotoxin genes *hlgA*, *hlgB*, *hlgC*, and *hla* and clonal complex (CC)-specific alleles of *lukAB* (either CC8 or CC30, except for two patient isolates that had uncategorized *lukAB* types). All strains also had genes for nuclease I and SspB, while 82.5% (33/40) had the gene for staphylokinase; these represent a group of genes present in the ϕSa3 phage that is associated with a loss of beta toxin (*hlb*) gene integrity. As expected, there was variability among the genes that reside on mobile genetic elements and phages, such as *pvl* and *lukED*, and, especially, superantigen and superantigen-like genes such as *seg*, *sei*, *sem*, *sen*, *seo*, and *seu*. Collectively, our data indicate that SSTI patients were infected with a diversity of *pvl*-containing MSSA and CA-MRSA strains, among which CA-MRSA strain USA300 represented the dominant clone (Table S2).

### Features of innate immune responses of SSTI patients.

At the time of recruitment, neutrophilic leukocytosis was common among the *S. aureus*-infected patients (mean, 7.6 k ± 2.9 k/µl; median, 7.2 k; range, 2.8 k to 17.1 k) and was not found in uninfected controls (mean, 4.1 k ± 1.1 k/µl; median, 4.3 k; range, 2.3 k to 5.8 k) (Fig. S2A). All patients received a course of antibiotics, and at the 6-week follow-up visit (v2), white blood cell elevations had normalized (Fig. S2B and C). We also looked for changes in the levels of cytokines in the serum of patients from the acute phase to the convalescent phase (Fig. S2D). We found that serum levels of the proinflammatory cytokine interleukin-6 (IL-6) were often higher at acute-phase presentation (visit 1 [v1]) than at the 6-week time point. About half of the patients had increases in IL-2 levels in this 6-week period, while the others had decreased IL-2 levels or levels that remained the same. Changes in the levels of other serum cytokines, such as IL-22 and IL-17A, were also detected (Fig. S2D); however, unsupervised hierarchical clustering analysis did not uncover a common pattern associated with SSTIs in the samples analyzed, potentially due to human subject variability and differences in the severity of infection. Hence, *S. aureus* infection commonly elicited a systemic inflammatory response that abated with clinical resolution of the infection.

10.1128/mBio.02125-17.1 FIG S1  Murine immunization with *S. aureus* leucocidin subunits, and also infection with the *pvl-*negative *S. aureus* Newman strain, induces IgG that cross-reacts with structurally related leucocidin subunits. (a and b) C57BL/6 mice were immunized and boosted with single exotoxin subunits (LukS, LukE, HlgC, or LukD) or saline solution. Serum samples obtained 28 days postimmunization were diluted 1:100 in 1% BSA–PBS, and 100-µl volumes of samples were incubated overnight with antigen-coupled microspheres (Luminex, Austin, TX), washed, detected with 1:100 anti-mouse IgG–PE–1% BSA–PBS, washed, and analyzed on the Luminex 200 platform. Data corresponding to (a) percent amino acid identity and (b) median fluorescence intensity (MFI) are shown for the exotoxin antigens tested. (c) C57BL/6 mice were infected intravenously with the *S. aureus* Newman wild-type WT strain, a protein A-null (Δ*SpA*) mutant, or a triple mutant strain (Δ*SpA* Δ*sbi* Δ*lukED*) at a sublethal inoculum (1 × 10^6^) and then rescued with vancomycin (50 mg/kg of body weight) intraperitoneally (i.p.) at day 4 and day 5, with reinfection performed at day 10, and serum were obtained on days 9 and 14 for analysis. (d) A multiplex bead-based array was performed for infected-mouse serum samples from the experiment described for panel c using multiple serum dilutions in 1% BSA–PBS (1:600, 1:6,000), with MFI shown for the 1:600 serum dilution. Download FIG S1, PDF file, 1.2 MB. Copyright © 2018 Pelzek et al. 2018 Pelzek et al. This content is distributed under the terms of the Creative Commons Attribution 4.0 International license.

10.1128/mBio.02125-17.2 FIG S2  *S. aureus* SSTI patients showed changes in neutrophil counts and serum cytokines in the acute phase of infection that resolved in convalescence, suggesting a systemic innate immune response. (a) Acutely infected *S. aureus* SSTI patients (*n* = 53) were compared to other patients from the same cohort, including patients with Streptococcus sp. SSTI (*n* = 12), coagulase-negative staphylococcus SSTI (*n* = 12), and no-culture-growth SSTI (*n* = 19) and emergency room (ER) uninfected controls (*n* = 12). Grouped analysis was performed with analysis of variance (ANOVA) (Kruskal-Wallis test with Dunn’s multiple-comparison test) (*, *P* < 0.05; **, *P* < 0.01; ***, *P* < 0.001; ****, *P* < 0.0001). To generate the data shown in panels b and c, *S. aureus* SSTI patient absolute neutrophil counts were assessed at each visit (acute phase, 6-week-convalescent phase [*n* = 38], and 6-month-convalescent phase [*n* = 17]) and analyzed for longitudinal fluctuations by the use of Graphpad Prism by the Wilcoxon matched-pair signed-rank test. Lower and upper normal ranges for absolute neutrophil counts are marked by red lines corresponding to 1.8 and 9.0 k/µl, respectively. (d) Assessment of longitudinal modulation of serum cytokines in *S. aureus* SSTI patients. Sera from *S. aureus* SSTI patients (*n* = 39) at the acute-phase and 6-week-convalescent-phase time points were assayed using a LEGENDplex human T helper cytokine panel 13-plex kit (BioLegend), followed by four-parameter logistic curve fitting performed using BioLegend LEGENDplex Data Analysis software and extrapolation of values (in picograms per milliliter). These values were assessed for changes using the following equation: acute-phase values in picograms per milliliter − convalescent-phase values in picograms per milliliter. Data are presented as delta values in picograms per milliliter, with a positive value reflecting a higher cytokine concentration at the acute phase and a negative value reflecting a higher cytokine concentration at 6 weeks of convalescence. Calculations performed on the basis of the Pearson clustering method in R showed groups that had IL-22 and IL-13 values that were higher in the acute phase; IL-6 and IL-2 values that were higher in the acute phase; IL-2 values that were higher and IL-6 and IL-22 values that were lower in the acute phase; IL-6 values that were higher and IL-2, IL-22, and IL-13 values that were lower in the acute phase; interferon gamma (IFN-gamma) and IL-17A values that were lower in the acute phase; and IL-2 values that were lower in the acute phase; and some groups of patients with broad responses (99624, 79414, 10732, and 44570). Download FIG S2, PDF file, 0.4 MB. Copyright © 2018 Pelzek et al. 2018 Pelzek et al. This content is distributed under the terms of the Creative Commons Attribution 4.0 International license.

### Validation of multiplex bead-based *S. aureus* antigen array in murine models.

To survey *S. aureus* antigen-directed antibody responses, we developed a sensitive multiplex antigen bead-based array with a number of recombinant proteins encoded by *S. aureus* toxin genes (Table S3). This assay had a lower limit of detection of <1 ng/ml, as determined with monoclonal antibodies (data not shown), as previously reported (41). *S. aureus* antigens in this panel included leucocidin subunits and a family of structurally related bicomponent toxins that target and kill leukocytes and other host cell types (22, 42). Leucocidins are composed of “S” components that enable targeting of host receptors (LukS, LukE, HlgA, HlgC, and LukA) and “F” components that facilitate dimerization with the “S” component (LukF, LukD, HlgB, and LukB). Subsequent oligomerization of “S” and “F” dimers generates a functional toxin, which can cause host cell lysis through pore formation on the plasma membrane (42). Leucocidin SF (LukSF) and Panton-Valentine leucocidin (PVL) share ~70% amino acid sequence identity with leucocidin ED (LukED) and hemolysin-gamma AB or CB (HlgAB or HlgCB), while leucocidin AB (LukAB) and the single-component pore-forming toxin alpha hemolysin (Hla) are more distantly related (22).

10.1128/mBio.02125-17.9 TABLE S3  Antigens in multiplex panel for assessment of immunoglobulin binding. Download TABLE S3, PDF file, 0.2 MB. Copyright © 2018 Pelzek et al. 2018 Pelzek et al. This content is distributed under the terms of the Creative Commons Attribution 4.0 International license.

To validate the antibody-binding specificities detected in our multiplex assay, we assessed serologic IgG responses in groups of mice following immunization with individual leucocidin subunits. We found that immunization with LukS, LukE, or HlgC recombinant proteins induced IgG responses that exhibited cross-reactivity with structurally related “S” leucocidin components (Fig. S1A and B), suggesting that these structurally related factors share conserved immunogenic epitopes. Likewise, the IgG responses induced by immunization with recombinant LukD were cross-reactive with “F” components. In contrast, there was no induction of antibodies against the CC8 variant of LukAB or Hla antigens that possess only low levels of protein sequence homology to LukSF (Fig. S1B). We conclude from these data that immunization with individual leucocidin subunits can drive antibody recognition of structurally related subunits.

To further validate our methodologic approach for multiplex antigen-reactive serological surveys, we studied responses in mice, after systemic infection with a sublethal intravenous inoculum (1 × 10^6^) of *S. aureus* strain Newman, which were rescued with vancomycin postinfection (Fig. S1C). Whereas naive uninfected mice had no detectable IgG reactivity, the postinfection mice displayed inductions of class-switched antibodies against LukSF and other *S. aureus* leucocidins, despite the absence in the Newman strain of the *pvl* gene that encodes LukSF (Fig. S1D) (23, 43). In contrast, these antigen-reactive responses were not observed following infection with a Δ*spa* Δ*sbi* Δ*lukED* triple-knockout Newman strain that is devoid of the genes for the Ig-binding proteins (*spa* and *sbi*) and the LukED toxin. These results suggest that LukED expression during infection is responsible for the induction of antibodies targeting LukSF (Fig. S1D). Overall, we found that murine infection commonly induces cross-reactive leucocidin-directed antibodies, presumably by recognition of shared structural sequences from a common evolutionary ancestral toxin gene.

Overall, these studies confirmed that murine immune exposure to *S. aureus* leucocidins results in responses that include production of cross-reactive antibodies. We subsequently used this assay to study infection-associated antibody responses in humans.

### *S. aureus* antigen-reactive antibody levels are often elevated during clinical infection.

**FIG 1:**
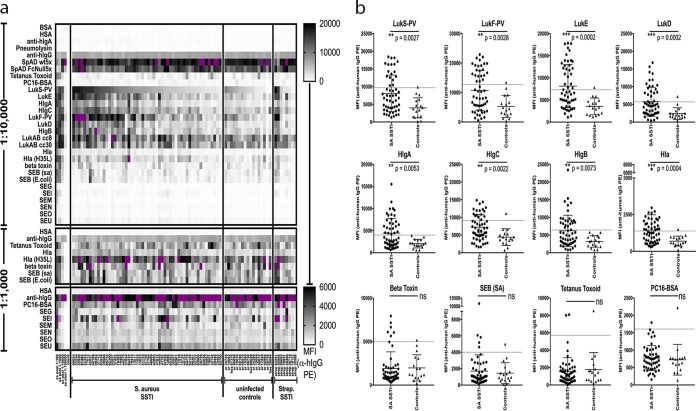
*S. aureus* antigen-reactive IgG levels are often higher in infected patients at acute-phase presentation than in healthy controls. Antigen-reactive IgG antibodies in patient serum samples were studied at multiple dilutions by multiplex bead-based antigen array for *S. aureus* SSTI patients (*n* = 54), uninfected controls (*n* = 17), and streptococcal SSTI patients (*n* = 8). (a) Data are presented as median fluorescence intensity (MFI) values for the 1:10,000 dilution, organized from highest to lowest within each group by reactivity to LukS, with data also shown for the 1:1,000 dilution for the antigen sets with lower IgG reactivity. Purple coloring denotes a signal above the upper limit of the scale. HSA, heat-stabile antigen. (b) Comparisons between *S. aureus* SSTI patients and uninfected controls are presented for the 1:10,000 serum dilution. The dashed line in each panel represents the mean plus 2 standard deviations for the uninfected control samples; the levels seen with SSTI samples above this threshold were considered to be elevated above control levels. Significance was determined using the Mann-Whitney test (ns, *P* > 0.05; *, *P* ≤ 0.05; **, *P* ≤ 0.01; ***, *P* ≤ 0.001.

To investigate human immune reactivity with secreted *S. aureus* proteins during and following infection with genetically diverse strains, we initially performed serological surveys. These studies documented that all infected patients, as well as control subjects, had detectable levels of IgG antibodies against a range of *S. aureus* exotoxin antigens (Fig. 1).

At acute-phase presentation, we observed that mean IgG antibody levels for infected patients were significantly greater than those for the uninfected controls for LukS (*P* = 0.0027), LukE (*P* = 0.0002), HlgA (*P* = 0.0053), HlgC (*P* = 0.0022), LukF (*P* = 0.0028), LukD (*P* = 0.0002), HlgB (*P* = 0.0073), Hla (*P* = 0.0004), and Hla (H35L) (*P* = 0.0192) but not for the tetanus toxoid (*P* = 0.7741) or PC (*P* = 0.4326) antigens that are not produced during these infections (Fig. 1b). However, we did not find significant elevations in antibody responses to toxins encoded by genes that are rarely represented in strain genomes that cause SSTI (Fig. S3), such as staphylococcal enterotoxins (SE) (*P* value for SEB = 0.6937, for SEG = 0.1638, for SEI = 0.0722, for SEM = 0.5628, for SEN = 0.1313, for SEO = 0.0916, and for SEU = 0.0745), or to beta toxin (*P* = 0.3454), which is typically inactivated by insertion of the ϕSa3 phage. We did not observe differences in levels of total serum IgG in patients compared with uninfected controls (data not shown). In addition, we analyzed IgG subclasses in serum samples from a limited subset of patients, and we found that infection-associated increases were not limited to a single IgG subclass (data not shown). Whereas IgG1 was dominant, there was marked heterogeneity between patients in the distribution of other subclasses, including various levels of antigen-reactive IgG2 and IgG4. Finally, we compared the groups of *S. aureus*-colonized and noncolonized SSTI patients at acute-phase presentation and found that the members of these groups showed no significant differences in antibody levels for any *S. aureus* antigen (data not shown).

10.1128/mBio.02125-17.3 FIG S3  Analysis of genomes for the presence of toxin genes in infecting and colonizing *S. aureus* isolates from human SSTI patients. Whole-genome sequencing was performed for infecting strains from 38 patients (*n* = 40 strains) and colonizing strains from 19 patients (*n* = 20 strains), and the results were analyzed by a custom BLAST-style alignment strategy (tBLASTn, translated nucleotides using a protein query) against a query amino acid sequence for each gene of interest, with NCBI protein accession numbers indicated in parentheses. The best match by percent amino acid identity within each genome is shown, with the exception of phenol-soluble modulins (PSM) alpha 2, 3, and 4, which were not detected in the tBLASTn strategy but instead with DNA-DNA BLAST. Download FIG S3, PDF file, 0.3 MB. Copyright © 2018 Pelzek et al. 2018 Pelzek et al. This content is distributed under the terms of the Creative Commons Attribution 4.0 International license.

Overall, serum antibodies were reactive with staphylococcal antigens commonly found in SSTI-causing strains. Our investigations found selective elevations in humoral levels of IgG antibodies to many secreted *S. aureus* toxins at the time that patients first presented with clinically apparent infection, consistent with earlier reports (36, 44, 45). There are a number of possible explanations for this (see Discussion).

### Variable humoral anti-*S. aureus* responses are induced in the long term by SSTI.

**FIG 2:**
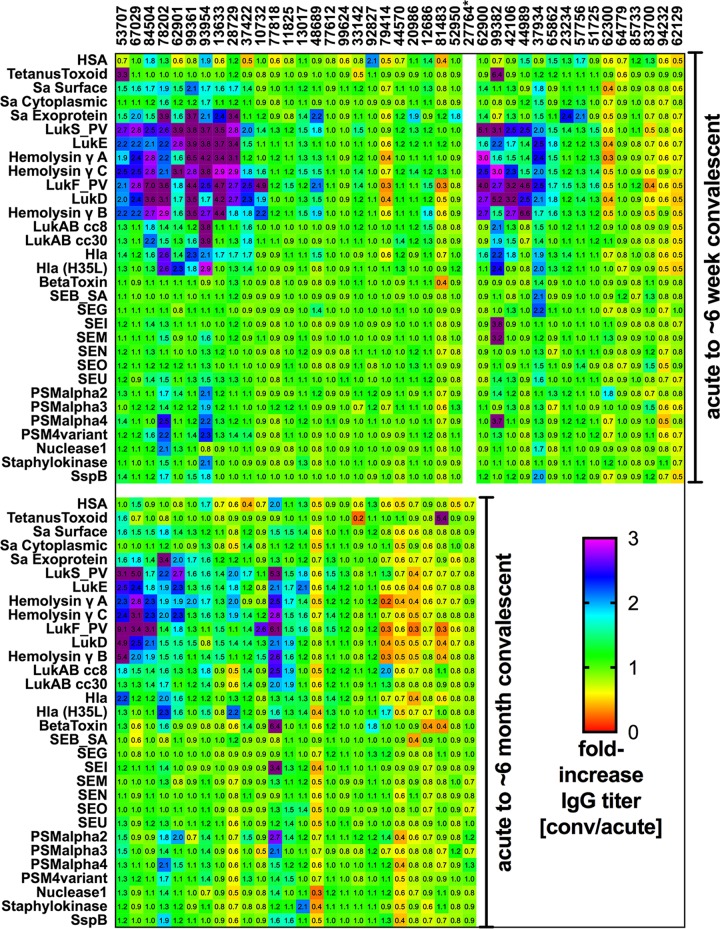
*S. aureus* skin and soft tissue infection induces longitudinal modulation of IgG levels against pore-forming exotoxin antigens in a subset of individuals. Patient serum samples were studied for IgG at 10-fold dilutions from 1:100 to 1:100,000 by multiplex antigen array. Median fluorescence intensity (MFI) values from all available visits were plotted for each individual for each antigen, and a high-throughput method was developed to assess antigen-reactive titers from the first visit (v1 [acute-phase infection]) to the second visit (v2 [at 6 weeks; *n* = 40]) and/or the third visit (v3 [at 6 months; *n* = 26]). Twenty-five subjects completed all visits. Fold changes in titer are presented here for each antigen, representing differences calculated with the following equation: convalescent-phase-sample value/acute-phase-sample value. The cutoff for an increased IgG response was set at a 2-fold increase from the acute-phase baseline, with relative decreases represented as values below 1. *, subject 27764 returned only for the 6-month visit.

To determine whether antigen-reactive antibody levels change in response to infection, we next compared IgG levels for each antigen in the serum of each patient at the acute-phase baseline to those from follow-up visits in our multiplex antigen array. Reactivity was tested with multiple serum dilutions, which enabled semiquantitative comparisons of antigen-reactive titers between visits (Fig. 2). At the 6-week follow-up visit, 55.0% (22/40) of patients had a 2-fold or greater increase in titer for at least one antigen-reactive IgG antibody response, and 37.5% (15/40) had increased titers for three or more *S. aureus* antigens. However, nearly half (i.e., 45.0%) did not attain the minimal (i.e., 2-fold) increase for any of the antigens tested, and these patients were designated nonresponders. Prior colonization status had no significant effect on infection-associated enhancement of the immune response for any *S. aureus* antigen studied (data not shown). From these studies, we concluded that many infected patients fail to mount broad increases in levels of anti-*S. aureus* antibodies against toxins encoded by infecting strains when examined after 6 weeks.

Of the subset of 25 *S. aureus* SSTI patients with serum samples obtained at all three time points, 48% (12/25) showed increased (i.e., 2-fold or greater) titers against one or more leucocidin subunits at 6 weeks. These patients, designated responders at 6 weeks, generally showed waning levels of antigen-reactive antibody when reevaluated at 6 months (v3) (8/12, 66%) (Fig. 2). Intriguingly, for some individual patients, increases in IgG titers against structurally related leucocidin subunits were observed to change in apparent synchrony, likely due to induction of cross-reactive antibodies (Fig. 2), for “S” components (patient 62901) and “F” components (patients 37422, 10732, and 81483) at 6 weeks and for “F” components (patients 78202, 62901, and 28729) at 6 months. In some cases, despite the absence of an exotoxin gene in the infecting strain, a humoral response for this antigen was nonetheless observed. For instance, two patients (99361 and 42106) who were infected with strains lacking the *lukED* gene had increased antibody responses to this toxin at 6 weeks (Fig. 2). Similarly, one patient (37934) whose isolate lacked the *lukSF* gene had an antibody response to both LukS and LukF subunits (Fig. 2).

These results therefore reiterate patterns and observations from our murine immunization and infection studies (Fig. S1 and S2), as antibody development may be driven by immune recognition following exposure to determinants also present in other structurally related leucocidin subunits. Collectively, these data suggest that antibody levels changed in response to the *in vivo* expression of determinants on structurally related leucocidin subunits. Nonetheless, our overarching observation was that it is generally uncommon for *S. aureus* SSTI to induce a long-lasting boost in humoral defenses that persists for even 6 months.

We also examined antibody levels for several other *S. aureus* antigens (Fig. 2) but found that infection-associated increases in IgG responses to Hla; Hla (H35L); LukAB (CC8 or CC30); beta toxin; phenol-soluble modulins (PSM) alpha 2, 3, and 4; nuclease I; staphylokinase; SspB; and staphylococcal enterotoxin (SE) proteins were observed in *S. aureus* SSTI only rarely. Notably, among the patients infected with isolates possessing genes for six of the SE proteins in our antigen array (patients 99361, 99624, 20986, 42106, 62300, and 83700), none exhibited persistent longitudinal antibody responses to these proteins. This suggests that, the postinfection humoral response to some other *S. aureus* antigens may be more limited than the more frequent occurrence of infection-associated increases in antibody titers corresponding to leucocidins.

Last, we wondered whether the detectable level of antigen-reactive IgG at presentation could be a determinant of longitudinal responses. Importantly, for both Hla (*P* = 0.04) and LukF (*P* = 0.05), we found that a higher IgG level at acute-phase presentation was inversely correlated with the fold increase in IgG titer at the follow-up visit (data not shown); we also observed trends for HlgA (*P* = 0.07), HlgB (*P* = 0.07), Hla H35L (*P* = 0.09), and SEB (*P* = 0.07). Therefore, for some *S. aureus* antigens, we conclude that an initially high level of antigen-reactive antibody at acute-phase presentation may limit the capacity to later increase these responses.

### Circulating anti-*S. aureus* memory B cells are abundant in SSTI patients.

We next investigated the representation of B cells reactive with *S. aureus* antigens. We used an approach that permits the assessment of a broad range of antigen specificities in individual culture wells (see Materials and Methods) (41). On the basis of the functional definition that only plasmablasts and plasma cells have the capacity to spontaneously secrete antibodies *in vitro*, we found that B cells spontaneously secreting IgG reactive with individual *S. aureus* antigens were detected only rarely and then only at low levels at the time of initial clinical presentation (i.e., 2/8 patients [25%]). B cells spontaneously secreting IgG reactive with *S. aureus* antigens were undetectable at the follow-up visit and in samples from control subjects (Fig. S4). Hence, we concluded that *S. aureus* antigen-reactive plasmablasts/plasma cells were uncommon in the circulation, even during active infection.

**FIG 3:**
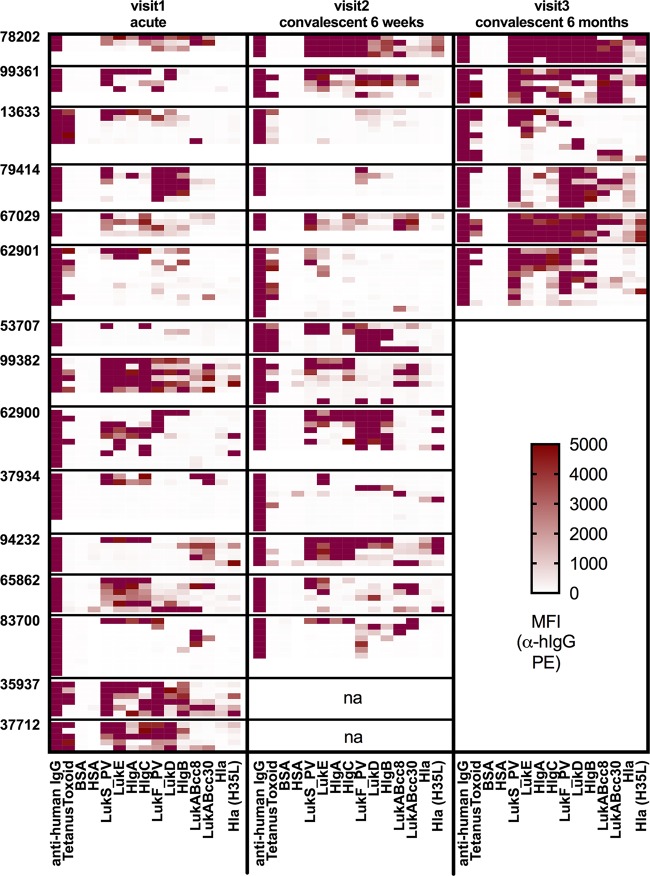
IgG-expressing memory B cells reactive with *S. aureus* exotoxin antigens are abundant in the peripheral blood of *S. aureus*-infected patients. PBMC (2 × 10^5^ cells/well) from *S. aureus* SSTI patients (*n* = 15) were cultured after isolation or following cryopreservation and thaw for 6 days with CpG2006/IL-21/sCD40L stimulation for acute-phase, 6-week convalescent-phase, and 6-month convalescent-phase samples. Supernatants containing antibodies were assessed at a 1:3 dilution for antigen-reactive IgG reactivity by bead-based multiplex assay. Each well is represented as one row, and data are reported as median fluorescence intensity (MFI) values. Purple coloring signifies MFI above the upper limit (>10,000 MFI).

10.1128/mBio.02125-17.4 FIG S4  *In vitro* induced PBMC have *S. aureus* exotoxin-reactive memory B cells in both *S. aureus*-infected and uninfected individuals, while only a subset of *S. aureus*-infected individuals exhibit spontaneous antibody secretion. PBMC (2 × 10^5^ cells/well) from *S. aureus* SSTI (*n* = 8) or uninfected emergency (ER) or operating room (OR) controls (*n* = 6) or non-*S. aureus* SSTI controls (*n* = 5) were cultured freshly after isolation for 6 days without (no stim) and with CpG2006/IL-21/sCD40L stimulation at the acute-phase time point (v1) and also at the 6-week convalescent-phase time point (v2) when available (with day of follow-up indicated in parentheses). Supernatants containing antibodies were assessed at a dilution of 1:3 for antigen-reactive IgG reactivity using a bead-based multiplex assay. Each well is represented as one row for each condition, and data are reported as MFI. Purple coloring signifies MFI above the upper limit (>10,000 MFI). On the basis of IgG secretion data, *S. aureus* antigen-reactive memory B cells were commonly detected both in freshly cultured PBMCs of eight *S. aureus* SSTI patients, as presented in this figure, and in the cryopreserved cells of an additional seven patients (see Fig. 3). Notably, five of six uninfected controls and two of five patients with nonstaphylococcal SSTI also had detectable levels of inducible memory B cells reactive with *S. aureus* exotoxins, demonstrating evidence of prior immune exposure. Download FIG S4, PDF file, 0.6 MB. Copyright © 2018 Pelzek et al. 2018 Pelzek et al. This content is distributed under the terms of the Creative Commons Attribution 4.0 International license.

To explore the reactivity and frequency of recirculating memory B cells against *S. aureus*, we assessed the capacity of quiescent lymphocytes from the bloodstream to undergo *in vitro* reactivation to become antigen-reactive IgG-secreting cells (ISC) (41, 46). In contrast to the results described above for cells spontaneously secreting Ig, we instead found that quiescent lymphocytes demonstrated robust inducible IgG responses to *S. aureus* exotoxin antigens, especially for subunits of the pore-forming leucocidins (Fig. 3; see also Fig. S4). These responses were detected both in samples from acute-phase presentation and in those from the 6-week and 6-month follow-up visits (Fig. 3). For SE proteins, most had only rare memory B-cell reactivity; among those, the most frequently detected were SEB and, to a lesser extent, SEI (Fig. S4 and S5). Thus, memory B cells against a range of *S. aureus* exotoxins were detectable, including common reactivity with leucocidins and hemolysins but with less frequency for superantigens.

10.1128/mBio.02125-17.5 FIG S5  IgG-expressing memory B cells reactive with staphylococcal enterotoxin antigens are generally infrequent in the peripheral blood of *S. aureus*-infected patients. PBMC (2 × 10^5^ cells/well) were cultured as described in the Fig. 3 legend. Each well is represented as one row for each condition, and data are reported as MFI. Results are shown for *S. aureus* SE proteins (superantigen and superantigen-like). Purple coloring signifies MFI above the upper limit (>10,000 MFI). Download FIG S5, PDF file, 0.3 MB. Copyright © 2018 Pelzek et al. 2018 Pelzek et al. This content is distributed under the terms of the Creative Commons Attribution 4.0 International license.

In a given subject, the mean antigen reactivity levels of IgG secreted from memory B cells *in vitro* significantly correlated with serum levels of IgG reactive at acute-phase presentation for LukF, LukD, HlgB, Hla, Hla (H35L), and SEB and at the 6-week follow-up for LukE, HlgA, HlgC, HlgB, Hla, Hla (H35L), and SEB (Table 1). Thus, quiescent memory B cells, rather than circulating plasma cells, predicted serum IgG antibody levels. Of note, in addition to the results seen with the *S. aureus*-infected subjects, quiescent memory B cells reactive against *S. aureus* exotoxins were also present in the circulation of uninfected controls (Fig. S4). However, this result was anticipated, owing to the known universality of prior *S. aureus* immune exposures among adults.

**TABLE 1:** *S. aureus* SSTI patient memory B-cell reactivity correlates with serological antibody levels for selected antigens

Antigen	Frequency of memory B cells at indicated serum dilution^a^					
	Visit 1 (acute phase; *n* = 15)			Visit 2 (6-week follow-up; *n* = 13)		
	1:1 × 10^3^	1:1 × 10^4^	1:1 × 10^5^	1:1 × 10^3^	1:1 × 10^4^	1:1 × 10^5^
BSA *p*	0.5377	0.8246	0.9142	0.5543	0.2964	0.4699
BSA *r*	*0.1717*	*−0.0626*	*0.0305*	*0.1798*	*0.3126*	*0.2181*
Tetanus toxoid *p*	0.2480	0.1832	0.2327	0.0673	0.0673	0.0673
Tetanus toxoid *r*	*0.3179*	*0.3628*	*0.3271*	*0.5275*	*0.5275*	*0.5275*
PC16-BSA *p*	0.1607	0.2212	0.5580	0.7454	0.7402	0.9022
PC16-BSA *r*	*0.3821*	*0.3357*	*0.1643*	*−0.0992*	*−0.1018*	*−0.0385*
Pneumolysin *p*	0.2321	0.0794	0.1637	0.4184	0.0184*	0.0520
Pneumolysin *r*	*0.3554*	*0.5069*	*0.4105*	*0.2561*	*0.6772*	*0.5790*
LukS *p*	0.7337	0.7435	0.9031	0.3633	0.5053	0.6167
LukS *r*	*0.0964*	*0.0929*	*0.0357*	*0.2747*	*0.2033*	*0.1538*
LukE *p*	0.4190	0.1607	0.2708	0.0073**	0.0525	0.0553
LukE *r*	*0.2250*	*0.3821*	*0.3036*	*0.7198*	*0.5549*	*0.5495*
HlgA *p*	0.8626	0.7144	0.7630	0.0028**	0.0067**	0.0109*
HlgA *r*	*0.0500*	*0.1036*	*0.0857*	*0.7747*	*0.7253*	*0.6923*
HlgC *p*	0.2888	0.0861	0.1094	0.0221*	0.0073**	0.0067**
HlgC *r*	*0.2929*	*0.4607*	*0.4321*	*0.6374*	*0.7198*	*0.7253*
LukF *p*	0.0068**	0.0017**	0.0017**	0.5537	0.3939	0.3436
LukF *r*	*0.6786*	*0.7536*	*0.7536*	*0.1813*	*0.2582*	*0.2857*
LukD *p*	0.0035**	0.0170*	0.0186*	0.6428	0.4819	0.4257
LukD *r*	*0.7179*	*0.6143*	*0.6071*	*0.1429*	*0.2143*	*0.2418*
HlgB *p*	0.0195*	0.0148*	0.0110*	0.0850	0.0320*	0.0284*
HlgB *r*	*0.6036*	*0.6250*	*0.6464*	*0.5000*	*0.6044*	*0.6154*
LukAB CC8 *p*	0.6482	0.7337	0.6115	0.2316	0.0812	0.1299
LukAB CC8 *r*	*−0.1286*	*0.0964*	*0.1429*	*0.3571*	*0.5055*	*0.4451*
LukAB CC30 *p*	0.8828	0.6575	0.8025	0.1585	0.3172	0.5254
LukAB CC30 *r*	*0.0429*	*0.1250*	*0.0714*	*0.4154*	*0.2999*	*0.1926*
Hla *p*	0.0054**	0.0134*	0.0424*	0.0004***	0.0007***	0.0005***
Hla *r*	*0.6929*	*0.6321*	*0.5344*	*0.8516*	*0.8352*	*0.8462*
Hla H35L *p*	0.0297*	0.0349*	0.0506	0.0101*	0.0079**	0.0051**
Hla H35L *r*	*0.5679*	*0.5536*	*0.5179*	*0.6978*	*0.7143*	*0.7418*
Beta toxin *p*	0.4151	0.4934	0.8631	0.9739	0.9210	0.5431
Beta toxin *r*	*0.2473*	*0.2088*	*0.0550*	*−0.0140*	*−0.0350*	*−0.1958*
SEB *Staphylococcus aureus p*	0.0535	0.0431*	0.0738	0.0003***	0.0007***	0.0002***
SEB *Staphylococcus aureus r*	*0.5112*	*0.5326*	*0.4772*	*0.8611*	*0.8336*	*0.8776*
SEB r, *Escherichia coli p*	0.2650	0.1032	0.2592	0.1460	0.0525	0.1759
SEB r, *Escherichia coli r*	*0.3071*	*0.4393*	*0.3107*	*0.4286*	*0.5549*	*0.4011*
SEG *p*	0.3360	0.4281	0.9033	0.7277	0.4330	0.4067
SEG *r*	*−0.2889*	*−0.2393*	*0.0372*	*−0.1121*	*−0.2487*	*−0.2627*
Sei *p*	0.0930	0.1103	0.1517	0.0989	0.1474	0.3789
Sei *r*	*0.4890*	*0.4670*	*0.4231*	*0.5035*	*0.4476*	*0.2797*
SEM *p*	0.9243	0.7328	0.1111	0.5381	0.9960	0.9338
SEM *r*	*0.0303*	*0.1045*	*−0.4649*	*0.1961*	*0.0035*	*−0.0280*
SEN *p*	0.1413	0.3955	0.3856	0.7664	0.6192	0.4990
SEN *r*	*−0.4319*	*−0.2552*	*−0.2613*	*0.0979*	*0.1608*	*0.2168*
SEO *p*	0.9533	0.4961	0.4066	0.8692	0.6509	0.3789
SEO *r*	*−0.0193*	*0.2063*	*0.2503*	*−0.0560*	*0.1469*	*0.2797*
SEU *p*	0.9351	0.9782	0.8631	0.6509	0.6353	0.7160
SEU *r*	*−0.0275*	*0.0110*	*−0.0550*	*0.1469*	*0.1538*	*0.1189*

^a^ Boldface data indicate significant associations. Italic data indicate inverse correlations. *, *P* ≤ 0.05; **, *P* ≤ 0.01; ***, *P* ≤ 0.001.

In *S. aureus*-infected patients, the frequency of memory B cells (Fig. S6) was elevated for LukS at 6 months (*P* = 0.015), for LukF at 6 weeks (*P* = 0.039) and 6 months (*P* = 0.005), and for Hla at 6 months (*P* = 0.031) in comparison to the frequencies observed in a set of 15 controls (10 uninfected adults plus 5 patients with nonstaphylococcal SSTI), after correction for multiple comparisons. This result might have been expected, based on our findings described above that memory B cells correlated with serum IgG levels. While memory B-cell frequencies for LukSF and Hla were elevated at selected time points, there was no general trend of enhanced memory B-cell frequency following infection for other *S. aureus* antigens studied.

10.1128/mBio.02125-17.6 FIG S6  Quantification of the frequencies of induced supernatants with IgG reactivity to *S. aureus* exotoxin antigens. Induced supernatants were obtained as detailed in the Fig. 3 legend. The number of wells positive by multiplex assessment of IgG for each antigen for each patient was quantified, with a positive well defined as having an MFI signal value of >250. Results are presented for pore-forming toxins but not for superantigens due to spatial considerations. Download FIG S6, PDF file, 0.2 MB. Copyright © 2018 Pelzek et al. 2018 Pelzek et al. This content is distributed under the terms of the Creative Commons Attribution 4.0 International license.

In summary, memory B cells against *S. aureus* leucocidins and hemolysins were commonly present in both infected and control subjects but were less frequent for superantigens. The observed increase in the prevalence of memory B cells for toxins such as LukSF and Hla in convalescent-phase samples from patients with SSTI may reflect *in vivo* expansion during or following these *S. aureus* infections. However, plasmablasts and plasma cells that spontaneously secrete antibodies reactive with staphylococcal exotoxins were detected only rarely, suggesting that there may be inefficient *in vivo* generation or differentiation of memory B-cells into IgG-secreting cells during infection. Importantly, for a subset of *S. aureus* antigens, we observed a correlation between the levels of circulating antigen-reactive memory B cells in samples from a given individual and the concentration of serum antibody.

### IgG from memory B cells recognizes structurally homologous leucocidin subunits.

**FIG 4:**
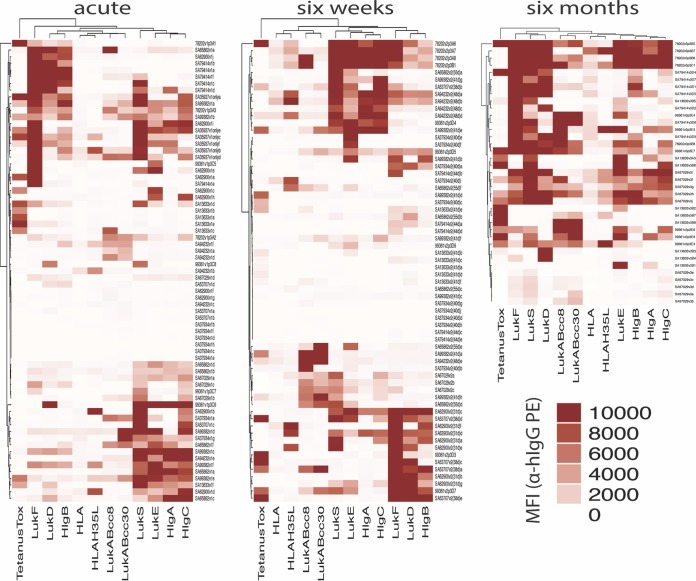
Induced memory B-cell cultures from *S. aureus* skin and soft tissue infection patients contain IgG that targets structurally homologous leucocidin components. Median fluorescence intensity (MFI) signals for antigen-reactive IgG from induced PBMC cultures from *S. aureus* SSTI patients at acute-phase and convalescent-phase visits were hierarchically clustered by the Euclidean method in R Studio using the program Complex heatmap. The Pearson clustering method provided similar results (not shown). Culture supernatants from acute-phase samples (visit 1, *n* = 12 patients), 6-week convalescent-phase samples (visit 2, *n* = 11), and 6-month convalescent-phase samples (visit 3, *n* = 5) were included in this analysis.

In our studies of *in vitro*-stimulated lymphocytes from *S. aureus*-infected individuals, we observed that memory B cell-derived IgG in individual culture wells generally reacted with either the “S” components or “F” components of the leucocidins but typically not both (Fig. 3). To assess this observation in greater detail, we used unsupervised hierarchical clustering to analyze common IgG reactivity patterns between patients (Fig. 4). We found that the data corresponding to memory B cell-derived IgG antibody binding from different individuals clustered, suggesting cross-reactivity among structurally homologous leucocidin subunits. Indeed, cross-reactivity was apparent between “S” components (LukS, LukE, HlgA, and HlgC) at both the acute-phase and 6-week time points and between “F” components (LukF, LukD, and HlgB) at 6 weeks of convalescence (Fig. 4), whereas there was a reciprocal paucity of memory B cell-derived antibodies recognizing single leucocidin subunits. However, clustering was not observed with LukAB or Hla, consistent with the greater divergence of their amino acid sequences than of those of the other more closely related “S” and “F” components of leucocidins in our panel (Fig. 4). This suggests that epitopes on LukAB and Hla antigens do not commonly elicit antibodies that cross-react with LukSF, LukED, HlgAB, or HlgCB. Overall, these results suggest that memory B cells are a common source of antibodies that are cross-reactive between structurally related leucocidin subunits.

### Serum and memory B-cell responses display cross-reactivity for “S”- or “F”-related sets of leucocidin subunits.

**FIG 5:**
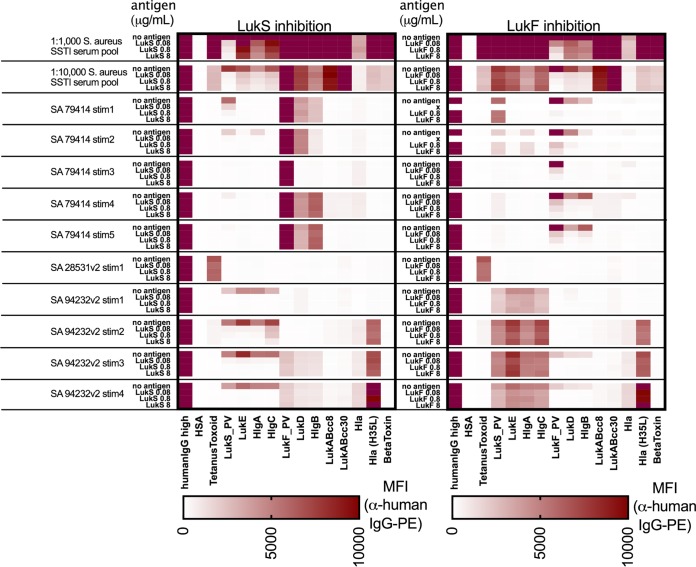
Cross-reactive antibodies in serum and induced memory B-cell supernatants against “S” or “F” leucocidin components can be inhibited by soluble antigen. A convalescent-phase *S. aureus* SSTI patient serum pool (*n* = 20 patients, 6 weeks convalescence) was diluted 1:1,000 and 1:10,000, and 90 µl diluted serum was treated with 10 µl of 1% BSA–PBS buffer (no antigen), LukF, or LukS and then assayed by multiplex bead-based array. For induced PBMC culture supernatants from two *S. aureus* SSTI patients (79414 and 94232) and one control supernatant with IgG reactive with tetanus toxoid (28531), 40 µl supernatant was added to 50 µl of 1% BSA–PBS and treated as described above. Median fluorescence intensity (MFI) values are shown.

To directly test whether the binding of anti-*S. aureus* IgG is commonly cross-reactive between different leucocidin subunits, we first evaluated the effects of preincubation with individual recombinant leucocidin proteins (LukF or LukS) as soluble competitors with serum IgG. With a pool of serum samples from 20 *S. aureus*-infected patients obtained at the 6-week follow-up visit, we found that preincubation with LukS at 8 µg/ml inhibited greater than 97% of the anti-LukS IgG reactivity, as well as 55%, 68%, and 57% of the reactivity with the structurally related “S” components LukE, HlgA, and HlgC, respectively (Fig. 5). In contrast, there was no significant reduction (i.e., <1%) of IgG reactivity with “F” components LukF, LukD, and HlgB. Conversely, preincubation with LukF inhibited more than 96% of LukF IgG reactivity, as well as 67% and 73% of reactivity with the related “F” components LukD and HlgB, respectively. In contrast, LukF preincubation resulted in less than 3% inhibition of binding with the “S”-related components. Overall, postinfection responses were dominated by antibodies that were cross-reactive between leucocidins of the same “S”- and “F”-related sets but not between those in the different sets, reflecting two dominant but distinct and coexisting patterns. Subunit-specific antibody reactivity was therefore relatively uncommon.

To evaluate whether recirculating memory B cells can produce antitoxin IgG with the same patterns of leucocidin subset cross-reactivity, we performed binding assays of IgG secreted *in vitro* by memory B cells from two *S. aureus*-infected patients. Notably, these studies also demonstrated that IgG binding to “S” components was inhibited by preincubation with LukS, but not by preincubation with LukF, as a soluble competitor (Fig. 5). Conversely, preincubation with titrated concentrations of LukF inhibited reactivity of IgG antibodies in wells with IgG-binding reactivity for “F” components, while LukS reactivity was not affected. We tested several memory B-cell supernatants from infected patients whose samples contained LukS/LukE cross-reactive antibodies for their capacity to neutralize LukED in a human neutrophil killing assay; however, we were unable to observe neutralizing activity (data not shown), which might have been expected due to the low level of total IgG produced in these cultures.

Taken together, we conclude that both memory B cells and serum antibody responses from patients with clinical infection commonly display cross-reactive binding for leucocidin subunits that share antigenic epitopes linked to common evolutionary genetic origins.

## DISCUSSION

Within the immune systems of healthy individuals, memory B cells convey host defenses with the capacity for highly specific recognition and accelerated clonal expansion in response to recurrence of exposure to a microbial pathogen. Ideally, infection should result in augmentation of the representation of memory B cells and long-lived plasma cells that contribute to stable and long-term humoral immunity (8, 18). Our investigations demonstrated that adults with *S. aureus* SSTI have expanded pools of circulating quiescent memory B cells that, upon *in vitro* reactivation, produce a plethora of antibodies that react with diverse *S. aureus* exotoxins, including especially high levels of reactivity with leucocidins (Fig. 3; see also Fig. S4 in the supplemental material), with greater heterogeneity of reactivity for other virulence factors included in our surveys (Fig. S5). Anti-*S. aureus* memory B cells were readily detectable in infected patients at clinical presentation and persisted well after resolution of infection. However, as these responses were also present in uninfected control subjects (Fig. S4), we postulate that these cellular responses initially arose from preceding *S. aureus* immune exposure (7).

Overall, our results argue against the notion that clinical *S. aureus* infection is responsible for a global functional defect in the generation of antigen-reactive memory B cells, and therefore complement findings of abundant T cell memory against *S. aureus* (47). Considering the ubiquity of serum class-switched antibodies, the existence of a large pool of memory B cells reactive to *S. aureus* exotoxins was not unexpected, as subclinical exposure to *S. aureus* is commonplace. Most importantly, our findings provide the first evidence of a relationship between the representation of staphylococcal antigen-reactive circulating memory B cells and serum antibody levels.

We observed a great range in the levels of serum antibodies between subjects at the time of initial clinical infection, while a high frequency of individuals showed few or no detectable variations in levels of antigen-reactive antibodies over time following resolution of the clinical infection. Even in the individuals in which antibody levels became elevated (i.e., >2-fold increases) at 6 weeks, most often for subunits of the pore-forming leucocidin subunits, antibody responses often dissipated by 6 months (Fig. 2). The genomes of the infecting *S. aureus* strains generally had many of the genes encoding the toxins in our antigen immunoassay arrays, including a high prevalence of *pvl* (Fig. S3), which is especially common in strains responsible for SSTI (39, 48). Our observations therefore suggest that in many individuals *S. aureus* infection is not sufficient to induce secondary recall responses that result in persistently elevated antibody levels (9, 34, 36, 49–51).

Our studies in murine models showed that both experimental *S. aureus* infection and immunization with a recombinant toxin can induce cross-reactive antibodies against structurally related sets of “S” or “F” leucocidin components (Fig. S1), in agreement with previous reports (23). Several of these toxins, i.e., LukSF, HlgCB, and LukAB, are now known to preferentially target surface receptors on distinct sets of human cells, suggesting coevolution of the host and this microbe (52, 53). Whereas some earlier studies of clinical cohorts described the presence of serum antibodies with cross-reactive binding specificities, others have found inconsistent results (54–59). Notably, we found that reactivated memory B cells of *S. aureus*-infected patients displayed recurrent dominant patterns of antileucocidin cross-reactivity, for sets of either leucocidin “S” or “F” components (Fig. 4 and 5). Additionally, the same cross-reactivity patterns were dominant in pooled convalescent-phase serum IgG from infected patients; therefore, little of the antibody reactivity was truly leucocidin subunit specific (Fig. 5). Despite the rarity of detectable *S. aureus* antigen-reactive circulating plasmablasts, our findings indicate that there is likely a direct functional relationship between antigen-reactive memory B cells and humoral IgG responses in each individual for the pore-forming toxins, including leucocidins and hemolysins.

We hypothesize that the presence of shared cross-reactivity patterns reflects the host’s capacity to recognize evolutionarily conserved motifs common among staphylococcal leucocidins. These vestigial commonalities may be the Achilles’ heel of leucocidins, as antibodies recognizing conserved determinants may also convey cross-neutralization functionality (58). Indeed, germ line-encoded VH region motifs may have been selected based on their capacity to neutralize IsdB, a staphylococcal virulence factor (60), and further studies are required to determine if this is true for leucocidins or other staphylococcal exotoxins. Both “S” and “F” components of the leucocidins have three important functional subdomains, including the rim that confers host cell targeting, the stem that inserts into the host cell membrane during pore formation, and the cap that covers hydrophobic residues in the stem domain. These features have been reviewed in detail by Spaan et al. (22), and further studies are required to determine whether dominant epitopes for human memory B-cell-derived antibodies localize to these domains. However, it has not been established that clones of such antibodies are alone sufficient to provide *in vivo* protection from clinical infection. Conversely, we speculate that the arms race between the human host immune system and *S. aureus* may lead B cells down a well-trodden and yet suboptimal immunologic path, where early and recurrent exposure drives the development of clonal responses that are restricted to immunodominant epitopes, in a manner reminiscent of original antigenic sin (61). Paradoxically, it has been reported that pediatric SSTI patients with the highest levels of *in vitro* LukSF neutralizing antibodies are not afforded protection from recurrent infections (24, 36). Hence, clonal focusing on cross-reactive epitopes (58, 60), linked to biased memory B-cell responses, may not best convey optimal *in vivo* protection but may instead aid the microbe to evade fully diversified host immune defenses (62).

Taken together, our studies have documented that, while the development of memory B cells is generally intact in patients suffering from and recovering from *S. aureus* SSTI (Fig. 3), active infection may result in inefficient secondary recall responses and suboptimal long-term humoral immunity (Fig. 2) (63). Spontaneous secretion of antibodies (normally attributed to plasmablasts) with antileucocidin reactivity was rare or absent during/following infection (Fig. S4). Further, in our serological studies, we observed elevated levels of antigen-reactive antibodies, especially against the leucocidins, at acute-phase presentation compared to the results seen with uninfected controls, akin to findings in earlier reports (64–66). Antibody responses to Hla, which are postulated to constitute a serological correlate of protection against future infection (67), were also less common in our cohort (Fig. 2). Notably, individuals with higher levels of anti-Hla and anti-LukF antibodies at acute-phase presentation also displayed a lack of subsequently enhanced antibody levels when reevaluated at follow-up visits. The explanation for this observation is unclear, although it may reflect (i) intrinsic host immune set points beyond which antibody levels will not further rise, (ii) the sequelae of remote immune exposures (22), (iii) strain-specific differences in toxin expression that are dependent on regulatory context (*agr*), or the *in vivo* bacterial burden that is associated with immune exposures to diverse staphylococcal antigens (iv) that may differ in their levels of local production or (v) that may vary in different individuals in a manner related to the periods of immune exposure during infection that preceded the initial clinical presentation and treatment.

Our studies had some limitations. For practical reasons, because of the large number of different toxins in an infecting strain, we did not generally assess the capacity of serum responses to functionally inactivate individual toxins. Prior reports documented neutralizing titers for individual toxin antigens in infected and control patients for PVL (36, 54), for LukAB (35), for Hla (49), and for staphylococcal enterotoxins (21). Also, initial studies of sorted memory (i.e., CD19^+^ CD27^+^ IgD^−^ IgG^+^) B cells, which were devoid of plasma cells, showed that stimulation was required for the induction of *in vitro* antibody production. Also, while we found that circulating staphylococcal antigen-reactive plasmablasts were at best rare, the detection of these end-differentiated antibody-secreting B-lineage cells may have been affected by practical concerns, as, despite our best intentions, it is possible that our timetable did not capture the peak representation of circulating plasmablasts. Furthermore, our limited studies of memory B-cell-secreted IgG antibodies failed to demonstrate the capacity for toxin neutralization, likely due to the low concentrations of induced antigen-reactive antibodies generated *in vitro* under the study conditions.

Germinal center reactions enable clonal selection of antigen-reactive B cells as well as development of expansions of both memory B cells and the plasma cells/plasmablasts that augment humoral defenses. Wilson and coworkers documented the paucity of staphylococcal antigen-reactive plasmablasts recoverable from the peripheral blood of patients with active *S. aureus* infections associated with different clinical syndromes during *S. aureus* infection (68). Their studies indicated that staphylococcal protein A (SpA), a virulence factor with dual Ig-binding specificities which was previously shown by our research group to have potent B-cell superantigen properties (20), was capable of non-immune variable-region-mediated Fab-binding interactions with 30% to 50% of human polyclonal blood B cells, which mediated context-dependent B-cell expansion and/or activation-induced death (19, 20, 69–71). Vilen and colleagues recently reported that SpA released during infection in murine models induced transient extrafollicular expansions of short-lived V_H_III-biased B cells (71), as a consequence of the *in vivo* production of this B-cell superantigen. Concurrently, there were great reductions in protective secondary anti-*S. aureus* antibody responses and the generation of long-lived antigen-specific plasma cells in bone marrow survival niches (71). It is currently unknown whether such pathways are also relevant to human infection.

All attempts to date to develop vaccines against *S. aureus* have been unsuccessful (30), and the basis of susceptibility to reinfection by *S. aureus* remains poorly understood (9, 34, 36, 49–51). Yet most humans show immunologic evidence of previous exposure to *S. aureus* antigens in the form of antibodies (6, 7, 32, 40, 72), and our studies showed that this includes diverse pools of immune memory B cells. For staphylococcal infection, adaptive immunity priming and higher levels of serum IgG antibodies have been reported to improve outcomes for severe invasive *S. aureus* infections (31, 67, 73), whereas lower antibody levels at clinical presentation reportedly are associated with higher rates of fatality (51), although this topic is controversial (24). Thus, while the functional consequences associated with the repertoire of memory B cells that arise in response to *S. aureus* exposure and infection are incompletely understood, our documentation of the prevalence of these immune clones should enable the wide-scale isolation and functional characterization of monoclonal antibodies derived from memory B cells against *S. aureus* antigens. Our current findings therefore suggest a previously unsuspected practical methodologic approach for the molecular characterization of minimal epitopes on the staphylococcal toxins produced in the context of human infection and targeted by active B-cell immune responses.

In summary, our studies demonstrated there is commonly a substantial reservoir of memory B cells against a wide range of *S. aureus* exotoxins among patients with SSTI. We therefore conclude that there is no global defect for the generation of immune memory B cells against key *S. aureus* exotoxins in the general population and that there appears to be a relationship between the representation of circulating antistaphylococcal memory B cells and levels of IgG antibodies. And yet the fine specificity of these responses and the capacity to recognize epitopes in addition to those on structurally conserved sites may be important determinants of immune protection.

We therefore propose that efforts to optimize clinical protective vaccines should include development of leucocidin-derived components designed to drive host immune recognition beyond conserved epitopes. Further studies are required to more fully elucidate the most protective combinatorial set(s) of immunogens, defined based on the epitopes targeted and expressed, with the goal of inducing both broadly cross-reactive/cross-neutralizing and individual toxin-reactive protective B-cell responses to defend against *S. aureus* infection.

## MATERIALS AND METHODS

### Patient enrollment and sample collection.

Patients were enrolled at Bellevue Hospital or Tisch Hospital (New York University Medical Center, New York, NY), and samples were obtained (at acute-phase presentation and 6 weeks and 6 months later) under institutional review board (IRB)-approved protocols and with informed consent (see Table S1 in the supplemental material). Control subjects were enrolled at those sites and at the Hospital for Special Surgery (New York, NY) or the New York Blood Center. Infecting and colonizing *S. aureus* strains were also recovered (Table S2). Peripheral blood mononuclear cells (PBMC) were isolated by centrifugation, washed in phosphate-buffered saline (PBS), and either used when they were fresh or cryopreserved and stored in a liquid nitrogen freezer, and serum samples were stored in aliquots at −80°C.

### Protein expression and purification.

*S. aureus lukS*, *lukF*, *lukE*, *lukD*, and *lukAB* from clonal complex 8 (CC8) and CC30, gamma-hemolysin genes (*hlgA*, *hlgB*, and *hlgC*), *hla* (alpha toxin), *hlb* (beta toxin), *sspB*, *nucI* (nuclease I), *sak* (staphylokinase), *seg*, *sei*, *sem*, *sen*, *seo*, and *seu* open reading frames (ORFs) were expressed in a suitable *S. aureus* bacterial variant, as previously described (74). Sequences for *hlgA*, *hlgB*, *hlgC*, *lukE*, *lukD*, the CC8 variant of *lukAB*, and *hla* were from *S. aureus* strain Newman; *lukS*, *lukF*, *nucI*, *sspB*, and *sak* were from *S. aureus* strain FPR3757; the CC30 variant of *lukAB*, *seg*, *sei*, *selo*, *selm*, *selu*, and *seln* were from *S. aureus* MRSA252; and *hlb* was from *S. aureus* strain COL. The source of other antigens is described in Table S3.

### Bead-based multiplex array for assessment of antigen-reactive IgG.

A custom 47-plex protein array for the Magpix platform (Luminex, Austin, TX) was created by coupling a variety of highly purified recombinant *S. aureus* proteins, including exotoxins and superantigens as well as other bacterial antigens and control ligands, to individual microspheres (Table S3), adapting the manufacturer’s protocol.

For antigen-reactive IgG detection, 1,000 microspheres per analyte per well were premixed, sonicated, and added to 100 µl of diluted serum (1:100, 1:1,000, 1:10,000, or 1:100,000) or supernatants (1:3). IgG was detected with Fc-gamma specific anti-human IgG R-phycoerythrin (R-PE) (EBioscience) or with anti-mouse IgG (Fc-specific) *F*(ab′)2 PE (Jackson), and data were acquired on a Magpix instrument (Luminex) and reported as median fluorescence intensity (MFI) values.

### Longitudinal serologic IgG responses.

A high-throughput method was used to assess fold change of serological antigen-reactive antibody titers using a Python script. MFI values for each antigen for each patient visit were automatically plotted. A point within the linear range was selected for comparisons to titers of equivalent MFI levels for samples from other visits. We then calculated fold changes for each antigen for the visit 2 (v2) titer versus the v1 titer or for the v3 titer versus the v1 titer, with the cutoff for a positive response set at a level at least 2-fold greater than the value from the recruitment visit.

### Competition assays for IgG in serum and supernatants.

Serum diluted 1:1,000 and 1:10,000 in PBS–bovine serum albumin (BSA) (1%) was added to 96-well plates (Greiner Bio-One). For PBMC culture supernatants, 40 µl was added to 50 µl of PBS-BSA. Ten microliters of PBS-BSA buffer (no antigen), LukF, or LukS was added to wells and incubated 1 h at room temperature with shaking at 500 rpm. Plates were spun at 3,000 rpm, 90 µl of supernatant was transferred to a new 96-well plate, and binding to aliquots of antigen-coupled beads was then assessed, with data reported as MFI.

### *In vitro* PBMC stimulation for memory B-cell reactivation.

Freshly isolated or cryopreserved PBMC were cultured without or with stimulation using a cocktail optimized for reactivation of memory B cells (CpG2006, sCD40L, and IL-21) as previously described (41, 46), with the following modifications: in 48-well plates, 2 × 10^5^ cells per well were cultured in 200 µl of RPMI complete media. After day 6, 400 µl of warm media per well was added, plates were centrifuged, and supernatants were harvested, with IgG antibodies assessed by multiplex antigen array alongside a standard curve of pooled convalescent-phase *S. aureus* SSTI patient serum; values are reported as MFI. Quantification of positive wells per antigen was derived using a cutoff of 250 MFI for a positive well.

### *S. aureus* isolate *spa* typing and whole-genome sequencing.

For each *S. aureus* isolate, *spa* types were determined as previously described (75, 76). Clonal complexes (CCs) were assigned on the basis of *spa* typing results, using the multilocus sequence typing mapping database (http://www.mlst.net) or peer-reviewed reports (see Table S2 for a summary of strain isolates). Genomic DNA from *S. aureus* strain isolates was extracted as previously described (77, 78), and shotgun sequence data were obtained using 150-bp paired-end reads (Illumina) with ~100× genome coverage.

### Determination of exotoxin genetic diversity in strain isolates.

*De novo* assembly of shotgun sequence data used SPAdes (79) to produce draft genomes composed of approximately 20 contigs with N50 of >150 Kb for all isolates. For *S. aureus* antigens in our multiplex array, reference sequences for each gene were used to determine sequence diversity and variations that could affect immune epitope recognition. Briefly, BLAST alignments and a custom script were used to create a tabular list of each candidate antigen with its best match against each draft genome under analysis. Outputs included Blast E value, overall percent amino acid identity, number of gaps in the alignment, percent coverage of the reference protein, and presence of a stop codon within the coding sequence. Antigen genes were generally considered verified matches with the reference sequence at greater than 90% amino acid identity and 95% coverage.

### Statistical analyses.

Determination of elevated IgG levels for an antigen was based on a cutoff representing the mean value for the uninfected controls plus 2 standard deviations. For acute-phase levels of antigen-reactive IgG and memory B cells and for absolute neutrophil counts, differences between patient and control groups were analyzed using the Kruskal-Wallis test with Dunn’s test for multiple comparisons or Mann-Whitney tests. Spearman correlations were used to compare longitudinal fold changes (between the acute-phase and 6-week visits) from acute-phase IgG levels for each antigen and also to compare mean signals of induced memory B-cell-derived IgG antibodies to serologic IgG levels for each antigen. Correlations and comparisons were generally considered significant at a *P* value of <0.05.
