# Supplementary material for: Human Memory B Cells Targeting Staphylococcus aureus Exotoxins Are Prevalent with Skin and Soft Tissue Infection
Source: mBio. 2018 Mar 13;9(2):e02125-17. doi: 10.1128/mBio.02125-17 (PMC5850327; doi:10.1128/mBio.02125-17)
Supplement: TABLE S1 [file mbo002183773st1.pdf]

Table S1: *S. aureus* skin and soft-tissue infection patient data

		#	%	#	%
**Gender**		(n = 54)			
	Male	42	77.8%		
	Female	12	22.2%		
**Age (years) (Mean +/- SD)**[a]		45.0 +/- 13.2			
**Race/Ethnicity**		(n = 54)			
	White	17	31.5%		
	Black	9	16.7%		
	Asian	4	7.4%		
	Other	24	44.4%		
	Hispanic			14	25.9%
	Latino			8	14.8%
	Semitic, Mix			2	3.7%
**Current Living Situation**		(n = 54)			
	House	41	75.9%		
	Shelter/Group/Street	12	22.2%		
	No Answer	1	1.9%		
**Ever homeless**		(n = 54)			
	Ever Homeless	20	37.0%		
	Never homeless	33	61.1%		
	No answer	1	1.9%		
**Alcohol use**		(n = 54)			
	0-4 drinks/week	42	77.8%		
	5+ drinks/week	10	18.5%		
	no answer	2	3.7%		
**Drug use**		(n = 54)			
	Drug use (last 6 months)	18	33.3%		
	Drug use (last 1 year)	18	33.3%		
	Cigarette smoker	19	35.2%		
**Previous to acute visit**		(n = 54)			
	antibiotics	11	20.4%		
	antibiotics (previous 6 months)	14	25.9%		
	clinical drainage of abscess	3	5.6%		
	immunosuppressive agents	5	9.3%		
	hospitalized past 12 months	10	18.5%		
**History of previous *S. aureus* exposure (patient reported)**		(n = 54)			
	history of *S. aureus* MRSA infection	4	7.4%		
	Cared for person with skin infection	9	16.7%		
	Cared for person with 'MRSA' or 'Staph'	3	5.6%		
**Days infected before acute presentation (patient reported)**					
		(n = 54)			
	0-1 days	0	-		
	2-3 days	14	25.9%		
	4-6 days	17	31.5%		
	7-10 days	14	25.9%		
	12-15 days	5	9.3%		
	16+ days	3	5.6%		
	no answer	1	1.9%		
**Chronic Comorbidities**[b]		(n = 54)			
	None reported	27	50.0%		
	Chronic Co-morbidity	27	50%		
	Other Immunocompromising Disease	21	38.90%		
	Diabetes	9	16.7%		
	Other	4	7.4%		

**Other Immunocompromising Disease**[c,d,e]		(n = 21)			
	HCV	7	13.0%		
	HTN	4	7.4%		
	recurrent abscesses	5	9.3%		
**Abscess Location**[f]		(n = 54)			
	Head/neck	9	16.7%		
	Torso	7	13.0%		
	Groin/perineum/buttocks	8	14.8%		
	Upper extremity	19	35.2%		
	Lower extremity	14	25.9%		
**At acute visit**					
	pus draining spontaneously	30	55.6%		
**Result of visit**		(n = 54)			
	Discharged	37	68.5%		
	Hospital	6	11.1%		
	Observation in ER	10	18.5%		
	Detox	1	1.9%		
**Antibiotic prescribed after acute ER visit?**		(n = 54)			
	antibiotic	45	83.3%		
	no antibiotic	8	14.8%		
	Unknown	1	1.9%		
**Patient Follow-up visits**[g]		(n = 54)		range (days)	mean (days) +/- SD
	visit 2, convalescent, short-term	41	75.90%	(21-93)	41.9 +/- 10.9
	visit 3, convalescent, long-term	25	46.30%	(154-389)	198.5 +/- 46.9
**Infections between visits**[h,i]		**v1-v2**	**v1-v2 (%)**		
	Abscess	3	5.6%		
	Total	9	16.7%		
		**v2-v3**	**v2-v3 (%)**		
	Abscess	4	7.4%		
	Total	8	14.8%		
Footnotes:					
a. Uninfected controls (n = 23) were 91.3% male, with mean age 48.9 +/- 12.6 years old (range 28-75 years)					
b. Each of these chronic comorbidities was reported once: Eczema/chronic skin condition, chronic renal failure, congestive heart failure, HIV+ non immunosuppressed					
c. Each of these conditions was reported once: HPV, cancer, renal disease, hyperlipidemia, pulmonary fibrosis, glaucoma, coronary artery disease, asthma, diverticulosis					
d. Each of these conditions was reported twice: Epilepsy/seizures, Hypothyroidism, GERD, IVDU					
e. Patients 10000, 13017, 20986, 37712, 65862 had recurrent abscesses					
f. Three patients had multiple abscesses					
g. One patient did not attend the six-week visit, but did attend the six-month visit (189 days post-acute). This value was not used in calculating mean, standard deviation, or range.					
h. Each of these conditions was reported once between acute and six-week visit: Bumps, current infection never resolved, cellulitis, unknown, cold sore, herpes zoster					
i. Each of these conditions was reported once between six-week and six-month visits: cellulitis, fungal infection, pneumonia, folliculitis					
