# Supplementary material for: Human Memory B Cells Targeting Staphylococcus aureus Exotoxins Are Prevalent with Skin and Soft Tissue Infection
Source: mBio. 2018 Mar 13;9(2):e02125-17. doi: 10.1128/mBio.02125-17 (PMC5850327; doi:10.1128/mBio.02125-17)
Supplement: TABLE S2 [file mbo002183773st2.pdf]

Table S2: *S. aureus* infecting and colonizing isolates from SSTI patients

		# (%)
**Micro-plate growth of infecting isolate**		(n = 54 patients)
	Heavy growth	21 (38.9)
	Moderate growth	20 (37.0)
	Information not available	13 (24.1)
**Patients with *S. aureus* infection**[a]		(n = 54 patients)
	MRSA	32 (59.3)
	MSSA	22 (40.7)
	single *spa* type	48 (88.9)
	multiple *spa* types	6 (11.1)
		(n = 53 patients)
	pvl-positive	46 (86.8)
	spa type 1/t008	22 (41.5)
	USA300	13 (24.5)
**Patients colonized with *S. aureus***		(n = 54 patients)
	colonized (nares)	13 (24.1)
	colonized (groin)	12 (22.2)
	colonized (total)	25 (46.3)
		(n = 25 patients)
	single *spa* type	22 (40.7)
	multiple *spa* types	3 (12.0)
	MRSA	11 (44.0)
	MSSA	14 (56.0)
**Infection site *S. aureus* isolates**		(n = 63 isolates)
	MRSA	39 (61.9)
	MSSA	24 (38.1)
	pvl-positive	56 (88.9)
	spa type 1/t008	23 (36.5)
	USA300	15 (23.8)
**Colonizing *S. aureus* isolates**		(n = 29 isolates)
	MRSA	12 (41.4)
	MSSA	17 (58.6)
	pvl-positive	18 (62.1)
	spa type 1/t008	9 (31.0)
	USA300	6 (20.7)
**Colonizing / infecting isolate match**		(n = 25 patients)
	same *spa* type	16 (64.0)
	different *spa* type	9 (36.0)
Footnotes:		
a. For one patient, the infecting *S. aureus* isolate was lost.		
