# Supplementary figures and images for: Human Memory B Cells Targeting Staphylococcus aureus Exotoxins Are Prevalent with Skin and Soft Tissue Infection
Source: mBio. 2018 Mar 13;9(2):e02125-17. doi: 10.1128/mBio.02125-17 (PMC5850327; doi:10.1128/mBio.02125-17)

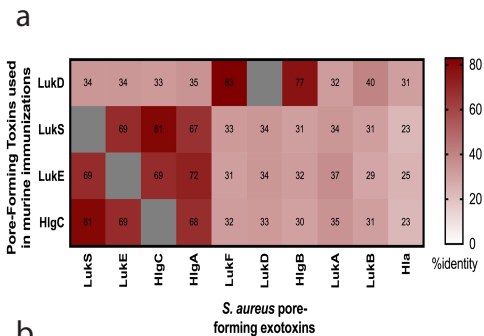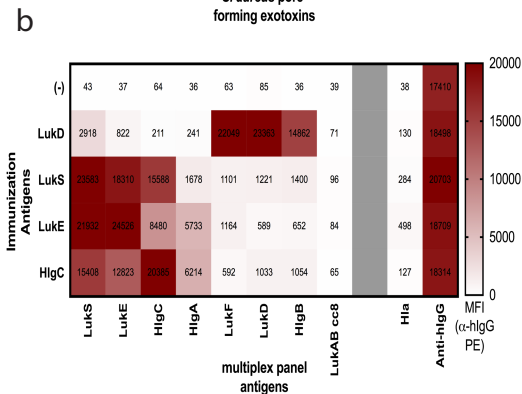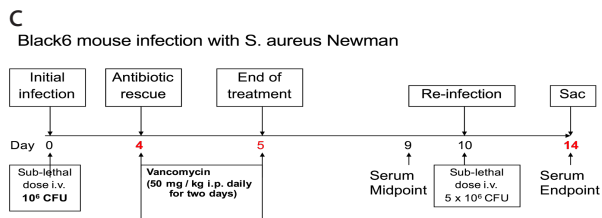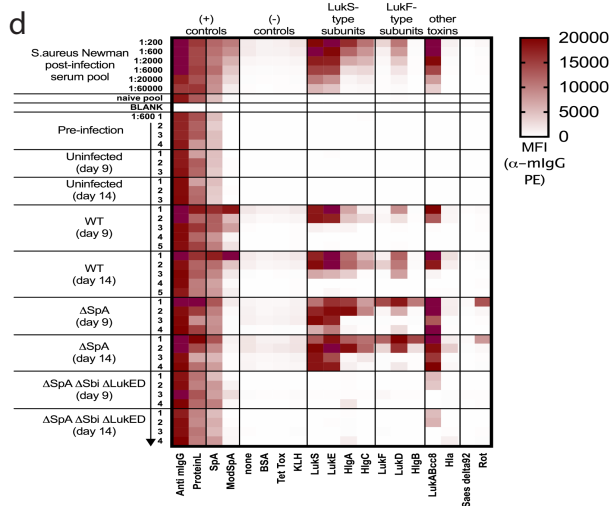

Supplement: FIG S1 [file mbo002183773sf1.pdf]

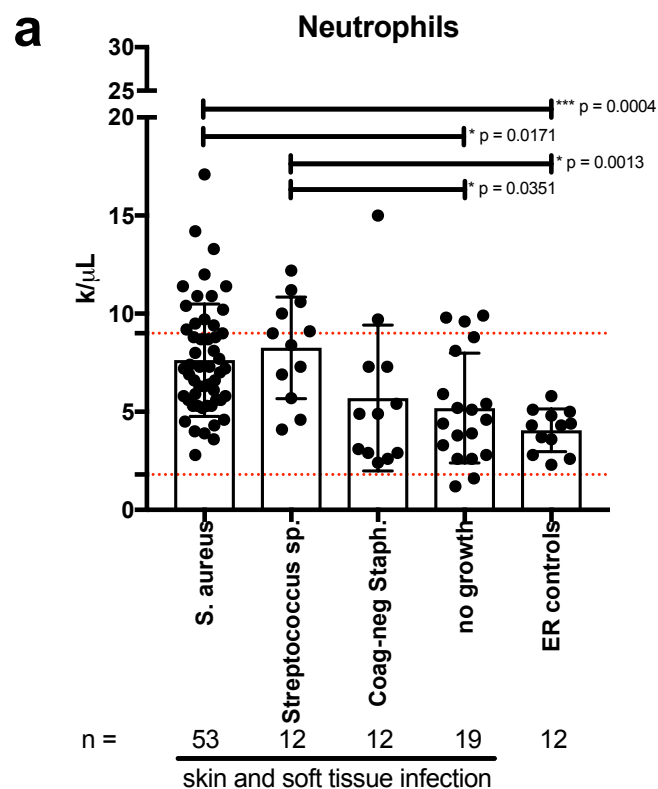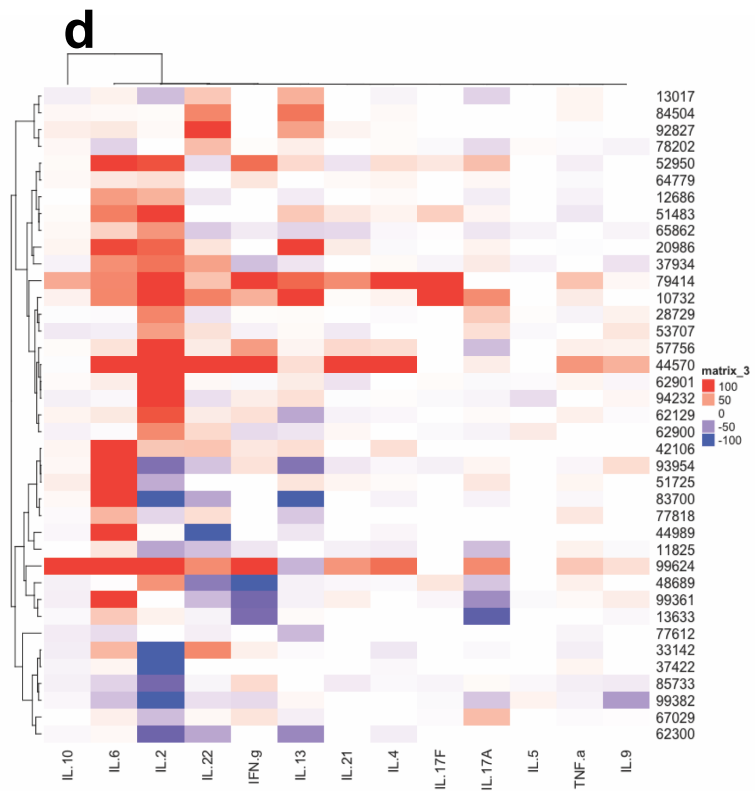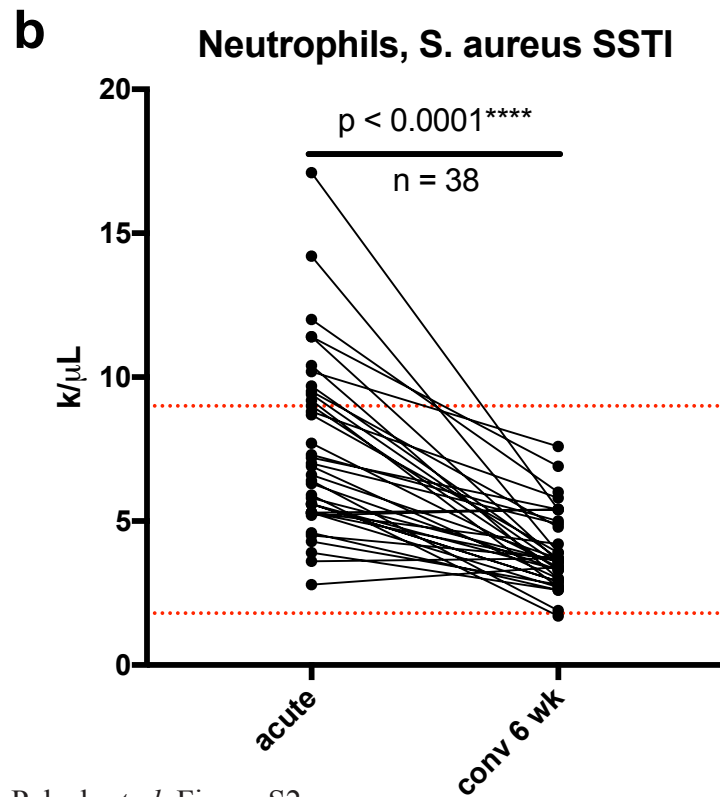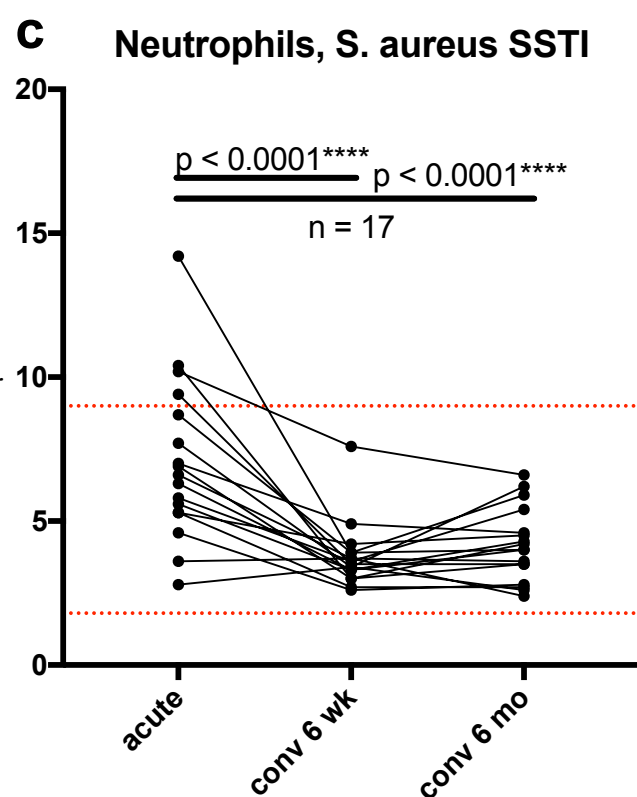

Supplement: FIG S2 [file mbo002183773sf2.pdf]

# infecting strains

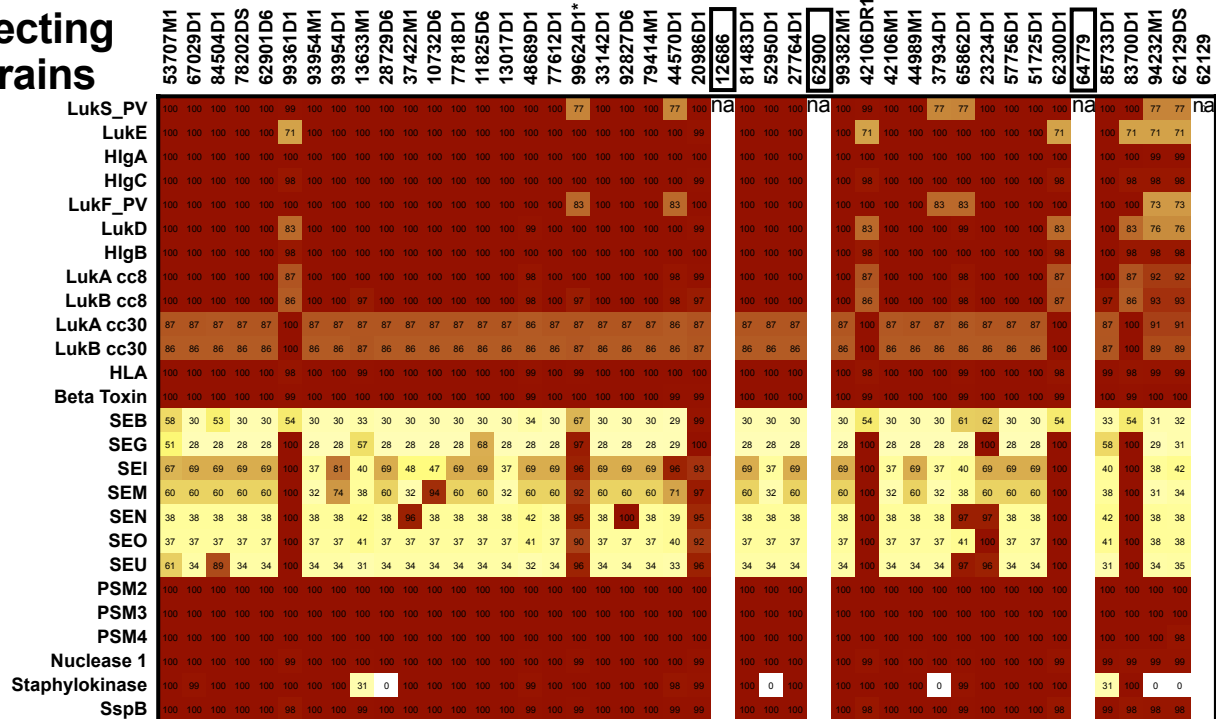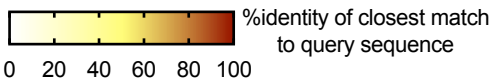

\* = coinfection with B-hemolytic Streptococcus

# colonizing strains

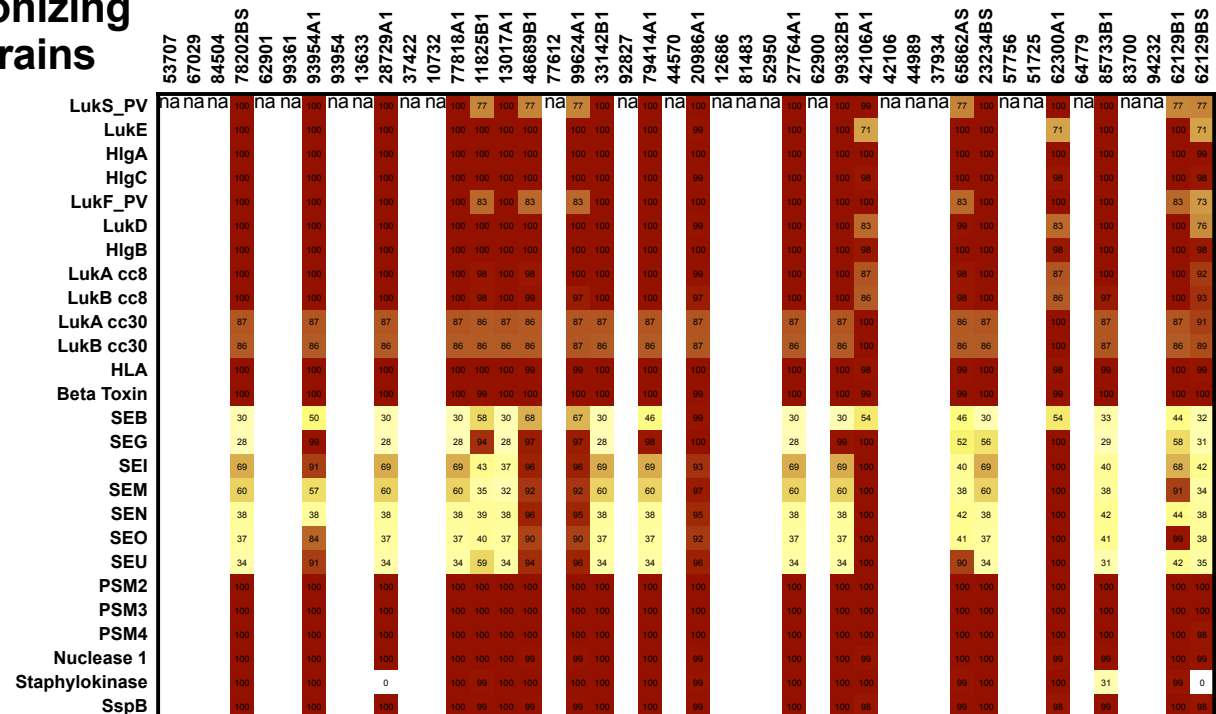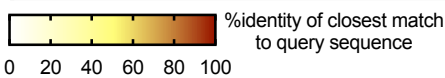

Supplement: FIG S3 [file mbo002183773sf3.pdf]

***S. aureus* SSTI**

**uninfected controls**

**non-*S. aureus* SSTI controls**

**PBMC (no stim)**      **PBMC (+ CpG2006/IL-21/sCD40L)**

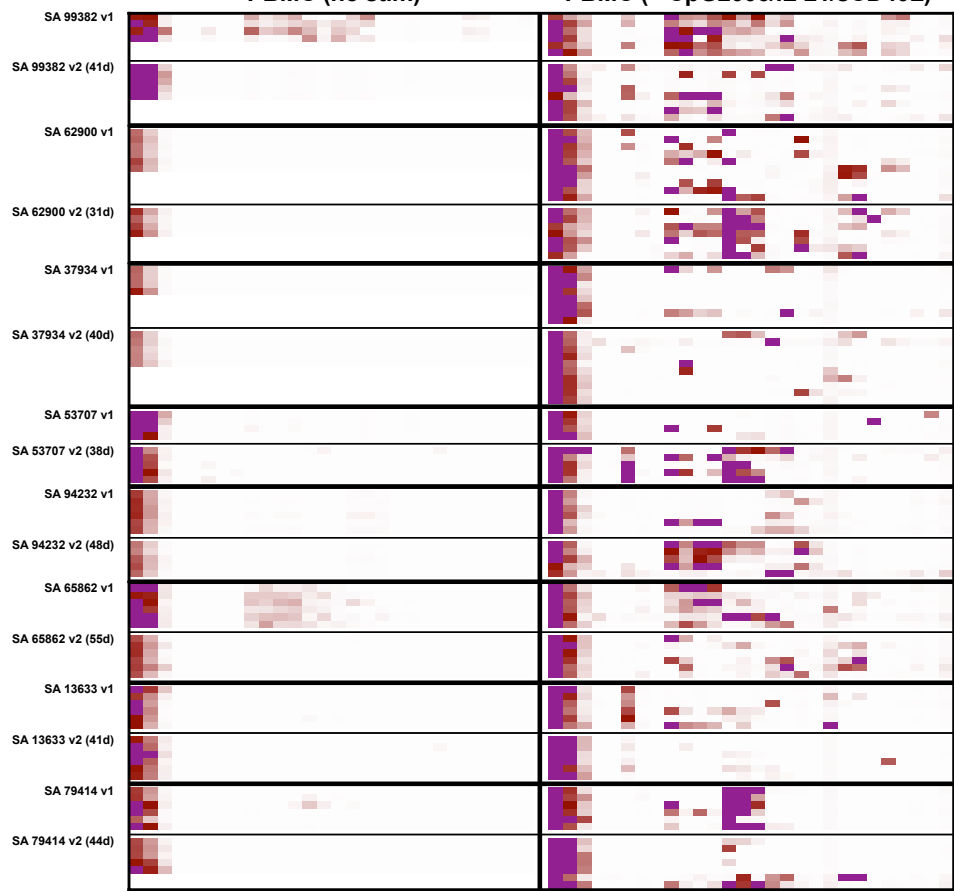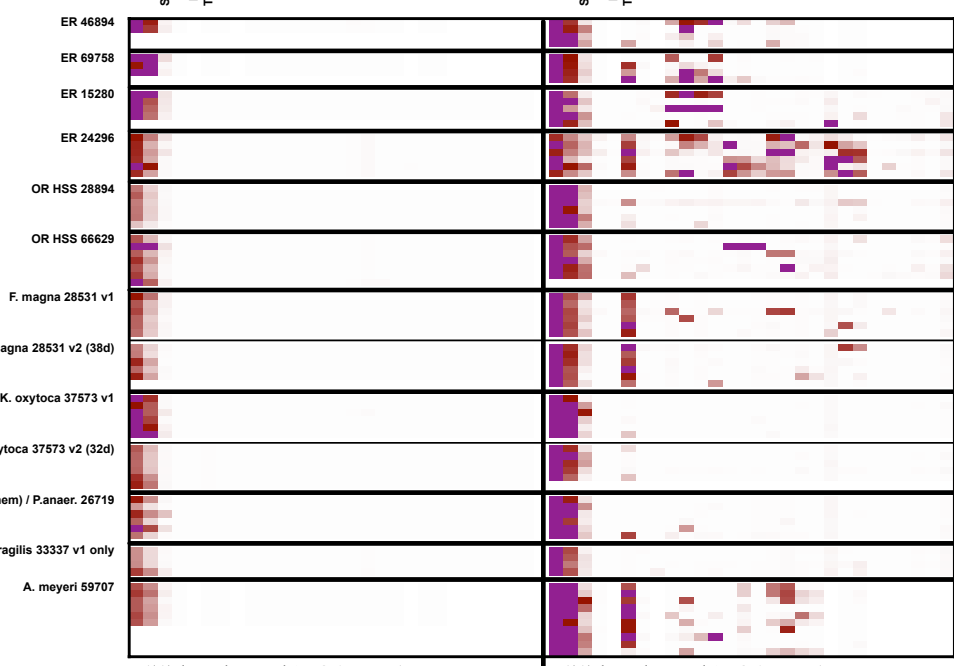

Supplement: FIG S4 [file mbo002183773sf4.pdf]

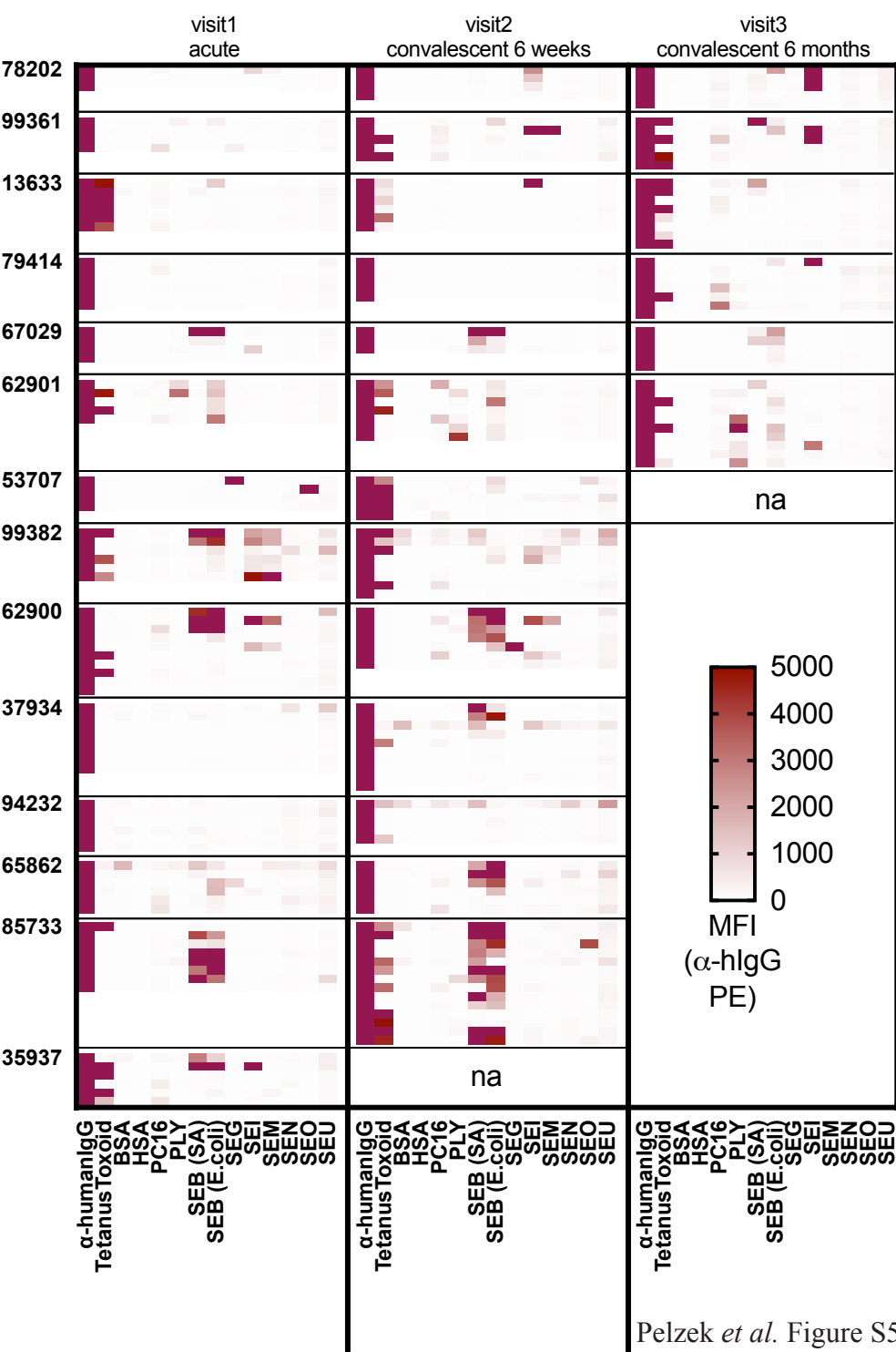

Supplement: FIG S5 [file mbo002183773sf5.pdf]

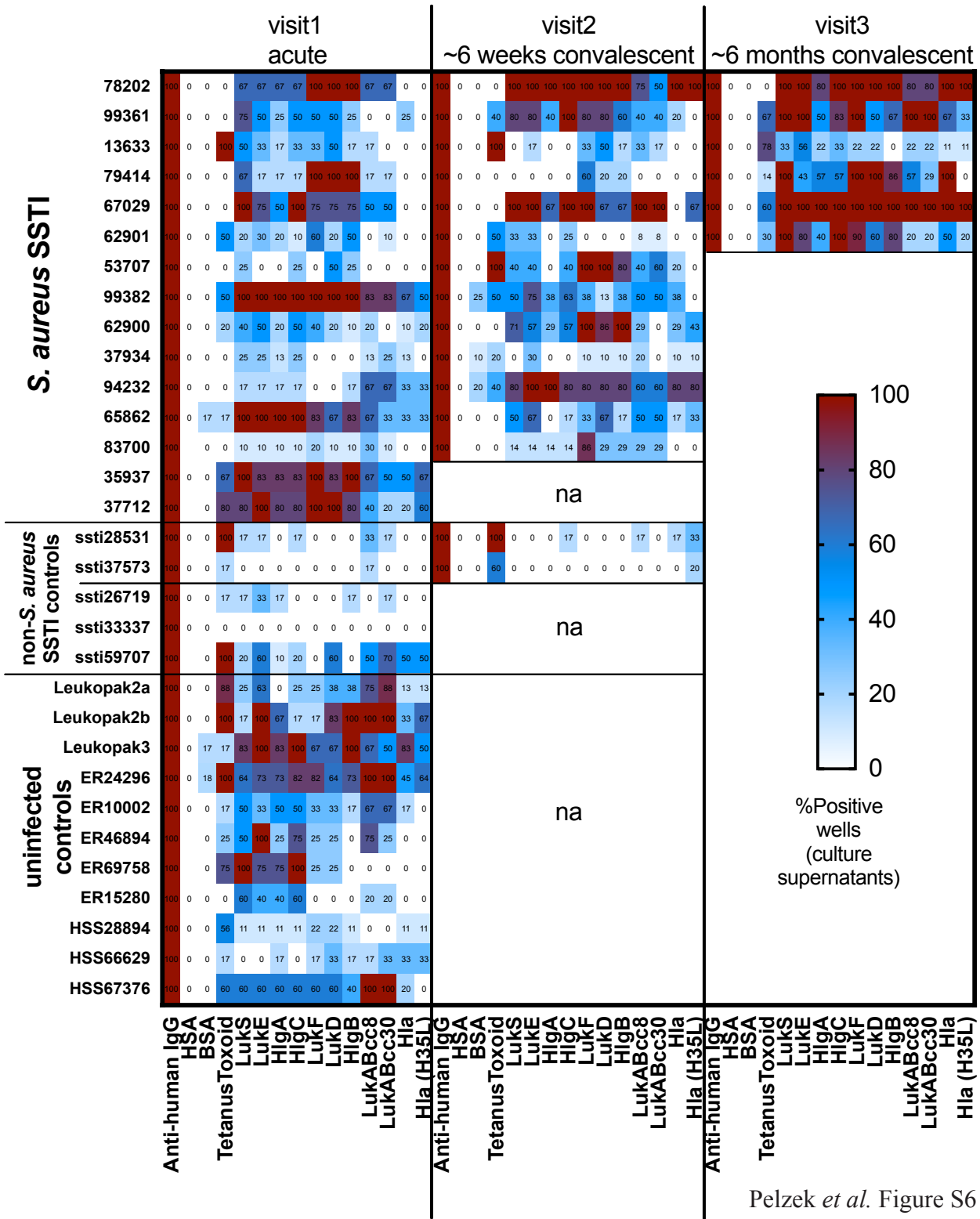

Supplement: FIG S6 [file mbo002183773sf6.pdf]
