# Supplementary material for: Human Memory B Cells Targeting Staphylococcus aureus Exotoxins Are Prevalent with Skin and Soft Tissue Infection
Source: mBio. 2018 Mar 13;9(2):e02125-17. doi: 10.1128/mBio.02125-17 (PMC5850327; doi:10.1128/mBio.02125-17)
Supplement: TABLE S3 [file mbo002183773st3.pdf]

Table S3. Antigens in multiplex panel for assessment of immunoglobulin binding

	Antigen	Abbreviation	Source	GenBank Locus[1]
**Positive controls**				
	Tetanus Toxoid	Tet Tox, TT	Enzo Life Sciences	
	Phosphorylcholine-16-BSA	PC16-BSA	Biosearch Technologies	
	Phosphorylcholine-12-BSA	PC12-BSA	Biosearch Technologies	
	Phosphorylcholine-4-BSA	PC4-BSA	Biosearch Technologies	
**Ig-binding**				
	anti-human IgG (Fc-gamma Specific)	anti-hIgG, α-hIgG	Jackson Immunoresearch	
	SpA domain D- WT (5x)[2]	SpAD-WT5x	G.J.S., D.N.H. (NYU)	
	SpA domain D- FcNull (5x)[3]	SpAD-FcNull5x	G.J.S., D.N.H. (NYU)	
**Negative controls**				
	Bovine Serum Albumin	BSA	Sigma Aldrich	
	Human Serum Albumin	HSA	Sigma Aldrich	
	Azobenzenearsonate	ABA	Biosearch Technologies	
***S. aureus* protein fractions**				
	surface extract		V.J.T. (NYU)	
	cytoplasmic extract		V.J.T. (NYU)	
	Exoproteins		V.J.T. (NYU)	
***S. aureus* Exotoxins**				
	Leukocidin S	LukS-PV	V.J.T. (NYU)	SAUSA300_1382
	Leukocidin E	LukE	V.J.T. (NYU)	NWMN_1719
	Hemolysin-gamma A	HlgA	V.J.T. (NYU)	NWMN_2318
	Hemolysin-gamma C	HlgC	V.J.T. (NYU)	NWMN_2319
	Leukocidin F	LukF-PV	V.J.T. (NYU)	SAUSA300_1381
	Leukocidin D	LukD	V.J.T. (NYU)	NWMN_1718
	Hemolysin-gamma B	HlgB	V.J.T. (NYU)	NWMN_2320
	Leukocidin AB cc8	LukABcc8	V.J.T. (NYU)	NWMN_1928, 1927
	Leukocidin AB cc30	LukABcc30	V.J.T. (NYU)	SAR2108, 2107
	Alpha Toxin (Alpha Hemolysin)	Hla	V.J.T. (NYU)	NWMN_1073
	Alpha Toxin (Alpha Hemolysin) H35L mutant	Hla (H35L)	V.J.T. (NYU)	
	β-Toxin (Beta Toxin)	β-Toxin	V.J.T. (NYU)	SACOL2003
***S. aureus* superantigens or Superantigen-like proteins**				
	Staphylococcal Enterotoxin B (chemically inactivated)	SEB (*S. aureus*)	BEI Resources	SACOL0907
	Staphylococcal Enterotoxin B (recombinant- inactive mutant, produced in *E. coli*)	SEB (r, *E. coli*)	BEI Resources	SACOL0907
	Staphylococcal Enterotoxin G	SEG	V.J.T. (NYU)	SAR1916
	Staphylococcal Enterotoxin I	SEI	V.J.T. (NYU)	SAR1919
	Staphylococcal Enterotoxin M	SEM	V.J.T. (NYU)	SAR1920
	Staphylococcal Enterotoxin N	SEN	V.J.T. (NYU)	SAR1917
	Staphylococcal Enterotoxin O	SEO	V.J.T. (NYU)	SAR1921
	Staphylococcal Enterotoxin U	SEU	V.J.T. (NYU)	SAR1918
***S. aureus* Phenol Soluble Modulins**				
	Phenol Soluble Modulin alpha 2	PSMa2	I.P.T. (Vanderbilt)	SAUSA300_0424.3
	Phenol Soluble Modulin alpha 3	PSMa3	I.P.T. (Vanderbilt)	SAUSA300_0424.2
	Phenol Soluble Modulin alpha 4	PSMa4	I.P.T. (Vanderbilt)	SAUSA300_0424.1
	Phenol Soluble Modulin alpha 4-variant	PSMa4-variant	I.P.T. (Vanderbilt)	
**Other *S. aureus* proteins**				
	Nuclease 1 -or- thermonuclease 1	Nuc1	V.J.T. (NYU)	SAUSA300_0776
	Staphylokinase	Sak	V.J.T. (NYU)	SAUSA300_1922
	SspB	SspB	V.J.T. (NYU)	SAUSA300_0950
	SaeS Δ92	SaeS Δ92	V.J.T. (NYU)	
	Rot	Rot	V.J.T. (NYU)	
**Other non-staphylococcal microbial antigens**				
	Pneumococcal polysaccharide 12	Pneumo ps12	ATCC	
	Pneumococcal polysaccharide 23	Pneumo ps23	ATCC	
	Poly N-acetyl glucosamine	PNAG	G.B. Pier (Harvard)	
	Pneumococcal Cell Wall Polysaccharide	Pneumo CWPS	Statens Serum Institut (Denmark)	
	S. pneumoniae pneumolysin	PLY	MyBiosource.com	
	S. pyogenes ArcA	ArcA	lab antigen stock	
	S. pyogenes superantigen	SP superantigen	lab antigen stock	

Footnotes:

1. Sequences obtained from reference strain genomes with accession numbers as follows: FPR3757 (SAUSA300; NC_007793.1), Newman (NWMN; NC_009641.1), MRSA252 (SAR; BX571856.1), COL (SACOL; NC_002951.2).
2. SpA domain D- WT (5x) is a concatamer of five copies of SpA domain D.
3. SpA domain D- FcNull (5x) is a concatamer of five copies of SpA domain D with three point mutations in each domain; in helix I, Q12K and Q13K, and in helix II N31A. These mutations destroy the Fc gamma-binding site and this construct therefore has only the non-immune binding of VH3-Fab containing IgG. References: Kim, Emolo, DeDent, Falugi, Missiakas & Schneewind (2012) Infect Immun 80**:** 3460-3470; and Cedergren, Andersson, Jansson, Uhlen & Nilsson (1993) Protein Eng. 6(4): 441-8.
